# *PPP1R3B* Suppresses Atherosclerosis by Promoting the M2 Polarization of Macrophages Through Glycogen Metabolic Reprogramming

**DOI:** 10.1002/advs.202506345

**Published:** 2025-09-23

**Authors:** Lin Shen, Junchao Yu, Weiqian Chen, Yanran Bi, Zhangyu Yang, Chenying Lu, Chengli Jiang, Yang Yang, Minjiang Chen, Jianhua Zou, Lingchun Lv, Xiaoyuan Chen, Jiansong Ji

**Affiliations:** ^1^ Zhejiang Key Laboratory of Imaging and Interventional Medicine Zhejiang Engineering Research Center of Interventional Medicine Engineering and Biotechnology Key Laboratory of Precision Medicine of Lishui City The Fifth Affiliated Hospital of Wenzhou Medical University Lishui 323000 China; ^2^ Department of Radiology Lishui Hospital of Zhejiang University School of Medicine Lishui 323000 China; ^3^ Department of Cardiology The Fifth Affiliated Hospital of Wenzhou Medical University Lishui 323000 China; ^4^ Clinical College of The Affiliated Central Hospital School of Medicine Lishui University Lishui 323000 China; ^5^ Departments of Diagnostic Radiology, Surgery, Chemical and Biomolecular Engineering, and Biomedical Engineering Yong Loo Lin School of Medicine and College of Design and Engineering National University of Singapore Singapore 119074 Singapore; ^6^ Nanomedicine Translational Research Program Yong Loo Lin School of Medicine National University of Singapore Singapore 117597 Singapore; ^7^ Theranostics Center of Excellence (TCE) Yong Loo Lin School of Medicine National University of Singapore 11 Biopolis Way, Helios Singapore 138667 Singapore; ^8^ Clinical Imaging Research Centre Centre for Translational Medicine Yong Loo Lin School of Medicine National University of Singapore Singapore 117599 Singapore; ^9^ Department of Pharmacy and Pharmaceutical Sciences National University of Singapore Lower Kent Ridge Road, 4 Science Drive 2 Singapore 117544 Singapore

**Keywords:** ASCVD, glycogen metabolic reprogramming, M2 macrophage polarization, PPP1R3B

## Abstract

Identifying targets that promote M2 macrophage polarization in the hypoxic plaque microenvironment is crucial for modulating immune metabolism and optimizing energy dynamics in atherosclerotic cardiovascular disease (ASCVD) treatment. The high phagocytic activity of M2 macrophages reduces foam cell formation. Their secretion of anti‐inflammatory cytokines enhances plaque stability, mitigating atherosclerosis progression. Through high‐throughput sequencing and multi‐omics bioinformatics analysis, protein phosphatase 1 regulatory subunit 3B (*PPP1R3B*) is identified as a key regulator linking glycogen metabolism to macrophage polarization. The integrated approach combined transcriptomic analysis of human atherosclerotic plaques (GSE57614) with RNA‐seq of *PPP1R3B*‐modulated macrophages, revealing its dual role. *PPP1R3B* induces anti‐inflammatory M2 macrophage polarization and maintains energy supply in plaques. Its absence accelerates plaque progression. *PPP1R3B* regulates M2 macrophage polarization and energy metabolism via phosphorylated STAT3 (p‐STAT3), which plays a dual role by activating anti‐inflammatory transcriptional programs through the PPAR‐γ/PGC‐1α/CD206 axis in the nucleus and enhancing glycogenolysis‐mediated metabolic activity via the p‐GSK‐3β/p‐PYGL/p‐GYS2 axis in mitochondria. *STAT3* plays a dual role in metabolic regulation and macrophage phenotype modulation. By orchestrating glycogen metabolic reprogramming, *PPP1R3B*‐induced M2 polarization presents a novel strategy for anti‐ASCVD drug development, with significant potential for clinical translation.

## Graphical abstract

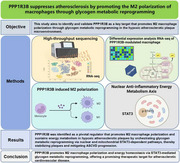

## 1 Introduction

Cardiovascular disease (CVD) is one of the leading threats to global health, accounting for high rates of illness and death.^[^ ^1^, ^2^ ^]^ ASCVD caused by atherosclerosis (AS) is the leading cause of morbidity and mortality in patients suffering from CVD.^[^ ^1^, ^3^ ^]^ The incidence of ASCVD continues to rise with the increasing prevalence of obesity and cardiac metabolic diseases, with its incidence and mortality increasing most rapidly in adults aged below 65,^[^ ^1^, ^4^ ^]^ Therefore, exploring the pathogenesis of ASCVD is vital to reducing the morbidity and mortality of patients with CVD.

AS is a chronic vascular inflammatory disease characterized by intimal injury, inflammatory cell recruitment, lipid accumulation, calcification, and plaque rupture.^[^ ^5^ ^]^ During the development of atherosclerosis, the polarization of macrophages (MΦs) showed significant differences at different stages. In the early stage of atherosclerosis, MΦs mainly exhibit M2 polarization and can secrete a large number of anti‐inflammatory factors (such as IL‐10, TGF‐β, etc.), which can alleviate the local inflammatory response and protect against the damage of arterial endothelial cells. This phenotype helps stabilize atherosclerotic plaque and prevent artery damage. Conversely, as the disease progresses and plaques become more unstable, MΦ polarization gradually shifts to the M1 phenotype. M1 MΦs have pro‐inflammatory effects and release a large number of inflammatory cytokines (such as TNF‐α, IL‐1β, IL‐6, IL‐12, etc.), thus promoting plaque instability and disease progression in the mid‐progression stage of atherosclerotic plaque formation (active plaque inflammation). Of course, in addition to M1 and M2 MΦs, there are specialized patch‐associated subtypes such as Mox, M4, M (Hb), and Mhem.^[^ ^6^, ^7^, ^8^, ^9^, ^10^ ^]^ Pro‐inflammatory M1 MΦs are characterized by increased glycolytic metabolism and impaired mitochondrial oxidative phosphorylation (OXPHOS). This metabolic shift leads to elevated secretion of inflammatory factors and increased reactive oxygen species (ROS) production, which disrupts the tricarboxylic acid (TCA) cycle and promotes cholesterol buildup and inflammation.^[^ ^11^, ^12^, ^13^, ^14^, ^15^ ^]^ In contrast, anti‐inflammatory M2 MΦs have a complete TCA cycle and enhanced OXPHOS, driven by peroxisome proliferator‐activated receptor gamma (PPARG/PPAR‐γ) and fatty acid metabolism, which inhibit proinflammatory pathways and promote anti‐inflammatory responses.^[^ ^16^, ^17^, ^18^ ^]^ At the same time, M2 MΦs engulf harmful substances such as oxidized low‐density lipoprotein (Ox‐LDL), this phagocytic activity that is essential for the stability of atherosclerotic plaques.^[^ ^7^, ^15^ ^]^ Moreover, studies have shown that activating the PPARγ signaling pathway has been proven to enhance the phagocytic capacity and anti‐inflammatory functions of M2 MΦs.^[^ ^19^, ^20^ ^]^

MΦs improve mitochondrial function by metabolic reprogramming from glycolysis to oxidative phosphorylation (OXPHOS) to support their functional phenotype. M1 MΦs rely on glycolysis to produce energy and promote inflammation, while M2 MΦs produce ATP via OXPHOS, supporting anti‐inflammatory and repair functions. M2 MΦs activate PPAR‐γ and PGC‐1α, enhancing mitochondrial biosynthesis and OXPHOS. *PPP1R3B* regulates glycogen metabolism, provides substrate for OXPHOS, and supports mitochondrial TCA cycle. p‐STAT3 promotes M2 polarization through the PPAR‐γ/PGC‐1α/CD206 axis in the nucleus and glycogenolysis and energy production through the p‐GSK‐3β/p‐PYGL/p‐GYS2 regulatory axis in the mitochondria. M2 MΦs reduce lactic acid accumulation, reduce inflammation and promote repair by enhancing OXPHOS, while maintaining mitochondrial membrane potential and reducing ROS production.^[^ ^21^ ^]^ This could ultimately enhance the quality of life for patients with ASCVD.

Protein phosphatase 1 regulatory subunit 3B (denoted as *PPP1R3B*), a key regulator of glycolipid metabolism reprogramming, is crucial in controlling MΦ polarization within human atherosclerotic plaques. As a regulatory subunit of protein phosphatase 1 (PP1), *PPP1R3B* plays a pivotal role in glycogen metabolism reprogramming by modulating the activity of glycogen synthetases and glycogen phosphorylases.^[^ ^22^ ^]^ Notably, *PPP1R3B* is highly involved in reprogramming glucose and lipid metabolism in ASCVD, highlighting its role in regulating glucose and lipid metabolism homeostasis.^[^ ^23^, ^24^, ^25^ ^]^ For example, Stender et al. found that the *PPP1R3B* expression correlates highly with glycogen/triglyceride/glucose and lipid metabolism homeostasis.^[^ ^25^ ^]^ As a major member of the PP1 family, *PPP1R3B* is a key target for treating metabolic diseases such as diabetes by regulating glucose and lipid metabolism homeostasis.^[^ ^26^ ^]^ Several studies have shown that overexpressing *PPP1R3B* can lower the risk of ASCVD by reprogramming glycolipid metabolism, enhancing glycogen synthesis, and reducing low‐density lipoprotein cholesterol (LDL‐C) levels.^[^ ^23^, ^24^, ^27^ ^]^ However, the specific mechanisms by which *PPP1R3B* orchestrates glycogen metabolic reprogramming to regulate MΦ polarization in ASCVD remain to be further explored.

**Scheme 1:**
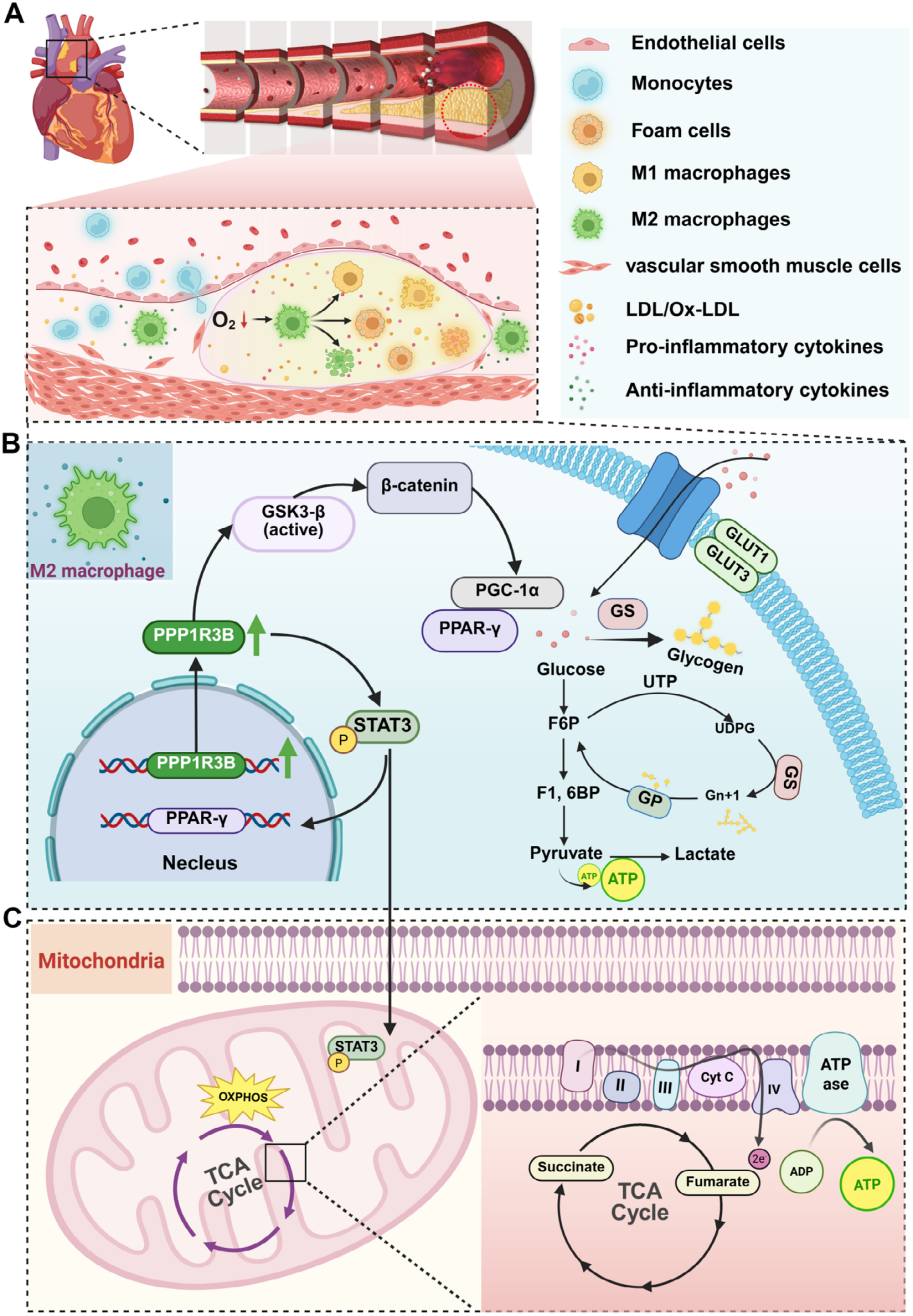
Schematic illustration of *PPP1R3B*‐mediated regulation of MΦ polarization and glycogen metabolic reprogramming in atherosclerosis. A) Hypoxic microenvironment and macrophage heterogeneity in atherosclerotic plaque. The top section illustrates the progression of atherosclerotic plaque development. It begins with endothelial injury and monocyte recruitment, followed by their differentiation into M1 (pro‐inflammatory) or M2 (anti‐inflammatory) MΦs, the formation of foam cells, and involvement of vascular smooth muscle cells. The central portion highlights the dynamic microenvironment within the plaque, emphasizing hypoxia, the coexistence of M1 and M2 MΦs, and the presence of both pro‐ and anti‐inflammatory cytokines. This provides essential context for understanding the functional relevance of MΦ plasticity during atherogenesis. B) *PPP1R3B*‐mediated transcriptional regulation of M2 polarization via nuclear STAT3 signaling. This section focuses on intracellular signaling within MΦs. Specifically, *PPP1R3B* expression is upregulated and activates key downstream regulators such as STAT3 and PPAR‐γ, both of which are crucial for driving M2 polarization and anti‐inflammatory gene expression. In addition, *PPP1R3B* modulates the GSK3β/β‐catenin and PGC‐1α signaling pathways, thereby bridging immune phenotype modulation with metabolic reprogramming. The final section details the metabolic consequences of *PPP1R3B* activation. It enhances glucose uptake via GLUT1/GLUT3 transporters, promotes glycogen synthesis through GS, and regulates glycogenolysis via GP. C) Mitochondrial STAT3‐driven glycogenolysis and OXPHOS activation in energy remodeling. These processes redirect intracellular glucose metabolism toward pyruvate and lactate production. Simultaneously, glycolytic intermediates enter the TCA cycle and mitochondrial OXPHOS, where *PPP1R3B*‐driven STAT3 activation enhances electron transport chain activity and ATP generation. This metabolic shift supports the energy requirements of M2 MΦs, contributing to anti‐inflammatory effects and plaque stabilization.

Here, we systematically analyzed the regulatory role of *PPP1R3B* in MΦ metabolic function and polarization within atherosclerotic plaques. These findings significantly advance our understanding of MΦ metabolic reprogramming and provide new therapeutic targets for AS treatment. *PPP1R3B* overexpression, by enhancing M2 polarization and metabolic function, modulates the plaque immune microenvironment, demonstrating notable anti‐atherosclerotic effects. Strong correlations between *PPP1R3B* expression, M2 markers (CD206 and ARG1), and p‐STAT3/PPAR‐γ/PGC‐1ɑ regulatory axis across models further support translational potential. Additionally, RNA‐seq and bioinformatics analyses reveal *PPP1R3B* cluster with immunometabolic genes (|r| > 0.9, *p* < 0.05), confirming their role as a pivotal metabolic regulator in atherosclerotic lesions and enhancing the clinical relevance (**Scheme** **1**).

## 2 Results and Discussion

### 2.1 *PPP1R3B* Loss in M2 MΦs Causes Atherosclerotic Plaques Formation in Human

Gene Expression Omnibus database was adopted to explore the key genes regulating the progression of the immune microenvironment in human atherosclerotic plaques. Analysis of the next‐generation sequencing (RNA‐seq) data for 36 human AS plaques in the GSE57614 dataset identified 4283 differentially expressed genes (DEGs) in human M1 and M2 MΦs (|log_2_(FC)| > 1 and adjusted *p* < 0.05), of which 1959 were upregulated and 2324 were downregulated (Figure S1A,B, Supporting Information).

**Figure 1:**
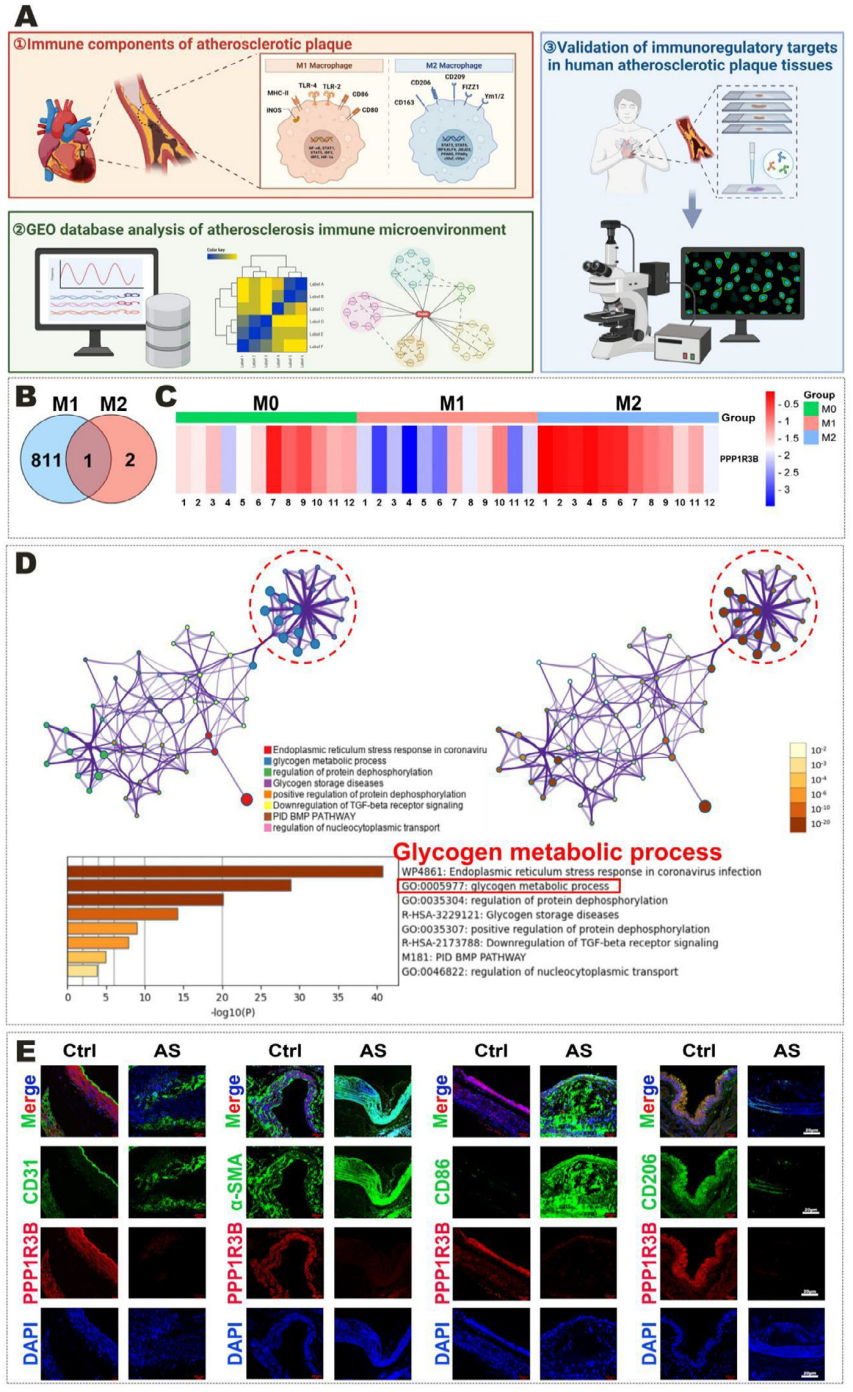
*PPP1R3B* is upregulated in M2 MΦs in atherosclerotic plaques. A) Schematic Diagram of Screening and Validation of Immunomicroenvironment Regulatory Targets in Atherosclerotic Plaques. B) Venn diagram of human AS‐associated MΦs (M1 and M2). C) Heatmap of *PPP1R3B* expression in human AS‐associated M0, M1, and M2 MΦs. M0 MΦs (unpolarized): GSM1385556 (M0‐1), GSM1385557 (M0‐2), GSM1385558 (M0‐3), GSM1385559 (M0‐4), GSM1385560 (M0‐5), GSM1385561 (M0‐6), GSM1385562 (M0‐7), GSM1385563 (M0‐8), GSM1385564 (M0‐9), GSM1385565 (M0‐10), GSM1385566 (M0‐11), GSM1385567 (M0‐12). M1 MΦs (pro‐inflammatory): GSM1515651 (M1‐1), GSM1515652 (M1‐2), GSM1515653 (M1‐3), GSM1515654 (M1‐4), GSM1515655 (M1‐5), GSM1515656 (M1‐6), GSM1515657 (M1‐7), GSM1515658 (M1‐8), GSM1515659 (M1‐9), GSM1515660 (M1‐10), GSM1515661 (M1‐11), GSM1515662 (M1‐12). M2 MΦs (anti‐inflammatory): GSM1515663 (M2‐1), GSM1515664 (M2‐2), GSM1515665 (M2‐3), GSM1515666 (M2‐4), GSM1515667 (M2‐5), GSM1515668 (M2‐6), GSM1515669 (M2‐7), GSM1515670 (M2‐8), GSM1515671 (M2‐9), GSM1515672 (M2‐10), GSM1515673 (M2‐11), GSM1515674 (M2‐12). D) Metascape analysis of (C). E) Representative immunofluorescence images of CD31, α‐SMA, CD86, CD206, and *PPP1R3B* staining in human aortic roots. The data are presented across three biologically independent samples. Scale bar: 20 µm.

Gene ontology (GO) and Kyoto Encyclopedia of Genes and Genomes (KEGG) enrichment analyses revealed that the DEGs regulating MΦs polarization in human atherosclerotic plaques were mainly involved in immune recognition and inflammatory signal pathways, such as caspase recruitment domain (CARD) binding that can respond to interferon‐γ, nucleotide oligomerization domain (NOD)‐like receptor signaling pathway, and the tumor necrosis factor (TNF) signal pathway (Figure S1C–E; Table S1, Supporting Information). Gene set enrichment analysis (GSEA) and ridge plots were used to visualize these DEGs, revealing that the inflammatory immune signaling pathway was significantly activated in M1 MΦs and inhibited in M2 MΦs (Figure S2A,B, Supporting Information). First, the relevant immune components in human plaque tissues were detected. Subsequently, the immune microenvironment regulatory targets were validated through analysis of the GEO database (**Figure** **1**A). Venn diagrams and heatmaps suggested that *PPP1R3B* is a key DEG regulating the polarization of MΦs in human plaques. Its expression was significantly higher in anti‐inflammatory M2 MΦs than in resting M0 MΦs and proinflammatory M1 MΦs (Figure 1B,C).

STRING database and Metascape analyses were used to explore the proteins that interact with *PPP1R3B* and their biological functions, whose results showed that *PPP1R3B* was closely related to glycogen metabolism‐related proteins, such as the PP1 family and glycogen synthase 1 (GYS1; Figure 1D; Figure S2C, Supporting Information). Combining GO/KEGG enrichment analysis with Cytoscape analysis further confirmed that *PPP1R3B* is closely related to protein phosphorylation, glycogen metabolism, and insulin resistance (Figure S2D,E, Supporting Information). This bioinformatic prediction (Figure 1D) was designed as an exploratory hypothesis‐generating step to build a regulatory network framework linking *PPP1R3B* with metabolic pathways.

Human MΦs derived from the THP‐1 cell line and human plaque tissues were used to further verify *PPP1R3B* expression in MΦs in human atherosclerotic plaques. First, M0, M1, and M2 MΦs were created from the human monocyte cell line THP‐1, and their protein expression was examined by western blotting. *PPP1R3B* expression was higher in M2 MΦs than that in M0 and M1 MΦs (Figure S3A,B, Supporting Information). Second, tissue immunofluorescence staining was used to explore the expression and localization of *PPP1R3B* in human atherosclerotic plaques and adjacent normal vascular tissues. *PPP1R3B* predominantly colocalized with the M2 MΦs biomarker mannose receptor C‐type 1 (MRC1/CD206), contrasting with its minimal colocalization with the endothelial cell biomarker platelet and endothelial cell adhesion molecule 1 (PECAM1/CD31), smooth muscle cell biomarker actin alpha 2 (ACTA2/α‐SMA), and M1 MΦs biomarker CD86 molecule (Figure 1E). Notably, CD206 and *PPP1R3B* expression in M2 MΦs was significantly reduced within atherosclerotic plaques, whereas CD86 expression in M1 MΦs was markedly increased (Figure 1E). Insulin was employed in this study due to its critical role as a master regulator of glucose and glycogen metabolism. Previous research demonstrated that insulin activates the AKT/GSK3 signaling pathway, which in turn modulates glycogen metabolism through the PP1/PPP1R3G axis. Since *PPP1R3B* is a key downstream effector of PPP1R3G‐influencing glycogen synthesis or breakdown‐we used insulin to investigate how *PPP1R3B* protein expression responds to different insulin concentrations and exposure times. Ultimately, stimulating the human THP‐1 monocytes with exogenous insulin increased *PPP1R3B* expression in a concentration‐ and time‐dependent manner (Figure S4, Supporting Information). In summary, these data indicate that downregulating *PPP1R3B* in M2 MΦs can exacerbate human atherosclerotic plaques.

### 2.2 *PPP1R3B* Induces M2 MΦ Polarization and Regulates their Immune Metabolism in Vitro

**Figure 2:**
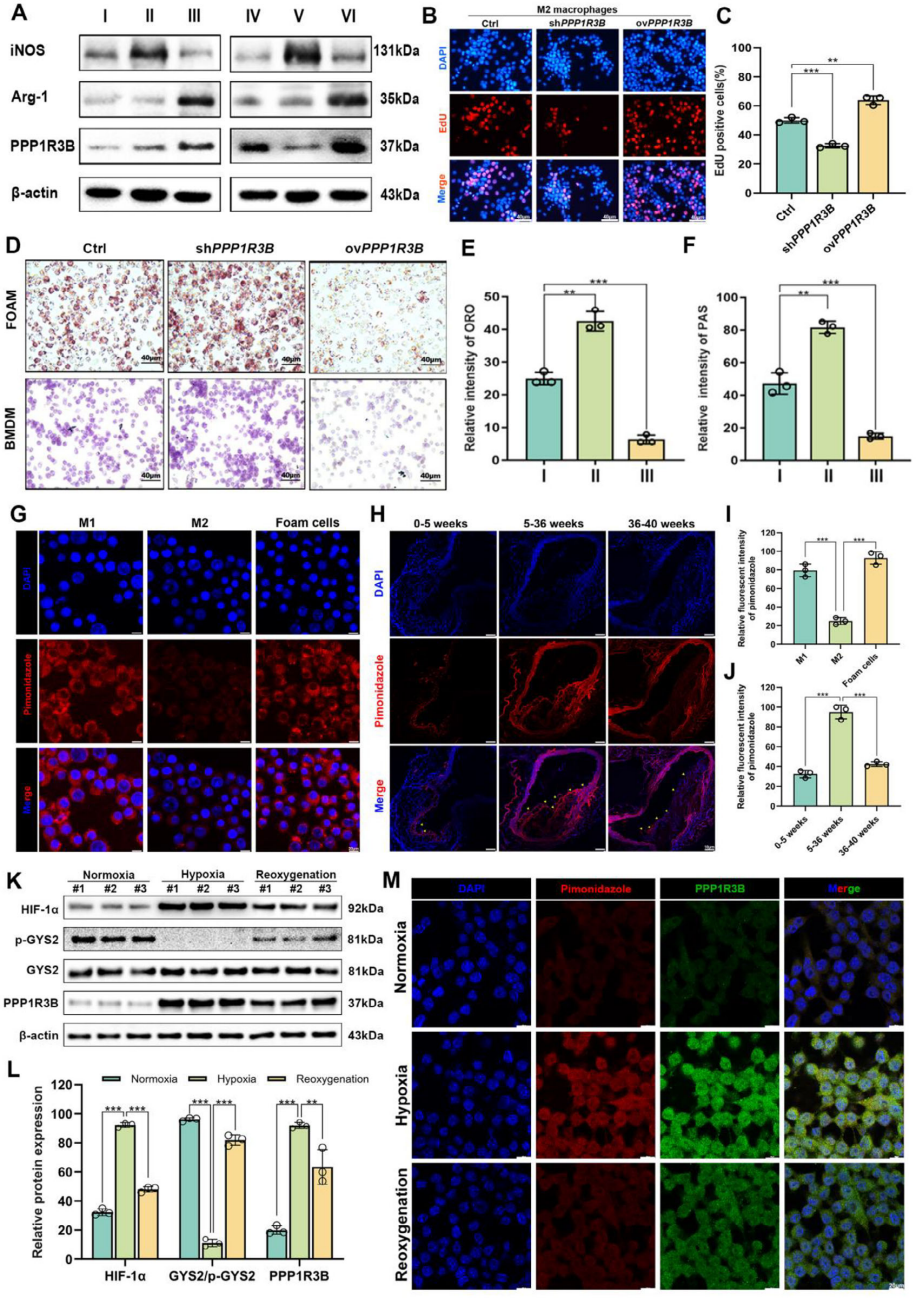
*PPP1R3B* regulates the immune metabolism of M2 MΦs in vitro. A) Western blot of *PPP1R3B*, iNOS, and ARG1 in MΦs from the different treatment groups; ACTB was used as the loading control. Group I: M0, Group II: M1, Group III: M2, Group IV: M0 + shCtrl, Group V: M0 + sh*PPP1R3B*, Group VI: M0 + ov*PPP1R3B* B) Representative fluorescence images and C) the corresponding quantitative analysis of EdU staining in M2 MΦs after different treatments. Scale bar: 40 µm. D) Representative images of ORO staining of foam cells regulated by *PPP1R3B* and PAS staining of glycogen metabolism in BMDMs regulated by *PPP1R3B*. Scale bar: 40 µm. A semi‐quantitative bar chart of E) images in control and sh*PPP1R3B* in Figure 2D and F) images in control and ov*PPP1R3B* in Figure 2D. Group I: Ctrl, Group II: sh*PPP1R3B*, Group III: ov*PPP1R3B*. G) Representative hypoxia staining images of M1, M2 MΦs, and foam cells. Scale bar: 20 µm. H) Representative images of hypoxia‐related immunofluorescence staining on cryosections of the aorta. Scale bar: 10 µm. I) Quantized statistical histogram of (G). J) Quantized statistical histogram of (H). K) Western blot analysis of HIF‐1α, GYS2, p‐GYS2 and *PPP1R3B* in M2 macrophages under normoxic, hypoxic, and reoxygenation conditions. L) Quantized statistical histogram of (K). M) Representative immunofluorescence images depict PPP1R3B expression (green) in M2 macrophages under normoxia, hypoxia, and reoxygenation. Scale bar: 20 µm. The data are presented as the mean ± SEM across three biologically independent samples. Significance: *p < 0.05; **p < 0.01; ***p < 0.001; ****p < 0.0001.

*PPP1R3B* overexpression (ov*PPP1R3B*) and knockdown (sh*PPP1R3B*) vectors were constructed to investigate the immunometabolic regulatory role of *PPP1R3B* in MΦ polarization related to AS (Figure S5, Supporting Information). The BMDMs were classified into the following groups: Group I (M0), phosphate‐buffered saline; Group II (M1), lipopolysaccharide; Group III (M2), interleukin 4 (IL4); Group IV, control small‐hairpin RNA (shCtrl); Group V, sh*PPP1R3B*; and Group VI, ov*PPP1R3B*. Western blotting revealed that overexpressing *PPP1R3B* in MΦs significantly increased the expression of arginase 1 (ARG1), a key immunoregulatory marker of M2 MΦs (**Figure** **2**A; Figure S6, Supporting Information). Conversely, knocking down *PPP1R3B* in MΦs significantly increased the expression of nitric oxide synthase 2 (NOS2/iNOS), a critical immunoregulatory marker of M1 MΦs (Figure 2A; Figure S6, Supporting Information).

*PPP1R3B* was found to promote the proliferative capacity of M2 MΦs, as evidenced by increased 5‐ethynyl‐2′‐deoxyuridine (EdU) incorporation and enhanced colony formation upon overexpression. Conversely, *PPP1R3B* knockdown significantly reduced both EdU‐positive cell percentages and clonogenic potential. These results indicate that PPP1R3B positively regulates M2 macrophage proliferation and survival (Figure 2B,C; Figure S7A,B, Supporting Information).

Cell cycle analysis revealed that overexpression of *PPP1R3B* in various MΦs types significantly extended the G2/M and S phases in M2 MΦs, promoting their proliferation (Figure S8A,B, Supporting Information). Furthermore, flow cytometry confirmed that overexpressing *PPP1R3B* in MΦs significantly promoted M2 polarization but inhibited M1 polarization (Figure S9, Supporting Information).

To elucidate the metabolic mechanisms by which *PPP1R3B* modulates MΦ function in atherosclerosis, we focused on its regulatory effects on glucose and lipid metabolism. Glucose and lipid metabolism of MΦs were examined to investigate the regulatory effect of *PPP1R3B* on lipid and glycogen metabolism in MΦs in AS. Oil Red O (ORO) staining revealed that overexpressing *PPP1R3B* significantly enhanced lipid efflux in foam cells, whereas knocking down *PPP1R3B* had the opposite effect (Figure 2D,E). Subsequent Periodic acid‐Schiff (PAS) staining demonstrated that *PPP1R3B* overexpression remarkably reduced glycogen accumulation within MΦs, while *PPP1R3B* knockdown led to a significant increase in glycogen deposition, highlighting its critical role in regulating glycogen metabolism within MΦs (Figure 2D,F). Immunofluorescence staining with Pimonidazole revealed varying levels of hypoxia among MΦ subtypes. M1 MΦs showed the strongest signals, M2 MΦs the weakest, and foam cells exhibited intermediate intensity, indicating distinct hypoxic burdens across phenotypes (Figure 2G,I). In aortic cryosections from *Apoe* ^−/−^ mice mice, Pimonidazole staining showed a progressive increase in hypoxia from early (0–5 weeks) to intermediate (5–36 weeks) and late (36–40 weeks) stages of atherosclerosis. Notably, pronounced hypoxic regions emerged during the intermediate stage and persisted thereafter, highlighting sustained hypoxic stress in advanced lesions (Figure 2H,J).

To investigate whether hypoxia induces metabolic reprogramming in M2 MΦs, cells were cultured under normoxic, hypoxic, and reoxygenation conditions. Under hypoxic stress, oxidative phosphorylation is known to be suppressed, forcing cells to adopt alternative energy‐producing pathways. Western blot analysis revealed that hypoxia markedly increased HIF‐1α and *PPP1R3B* expression, while significantly decreasing phosphorylated GYS2 (p‐GYS2) levels, indicative of activated glycogen synthase and a metabolic shift toward glycogen metabolism. Upon reoxygenation, HIF‐1α and *PPP1R3B* expression declined, and p‐GYS2 levels were restored (Figure 2K,L), suggesting a return to baseline oxidative metabolic activity. Immunofluorescence staining further confirmed elevated *PPP1R3B* expression in hypoxic macrophages, which diminished after reoxygenation, in line with increased pimonidazole staining under low‐oxygen conditions (Figure 2M). These findings indicate that hypoxic stress suppresses oxidative phosphorylation in M2 MΦs, while upregulating *PPP1R3B* expression to enhance glycogen metabolism, thereby achieving energy metabolic reprogramming.

Collectively, our findings indicate that *PPP1R3B* is a pivotal integrator of MΦ polarization and metabolic state, particularly under hypoxic conditions commonly encountered in intermediate‐to‐late stage atherosclerosis. By promoting M2 polarization, proliferation, and glycogen metabolism, *PPP1R3B* may contribute to the resolution of inflammation and maintenance of plaque stability. Targeting *PPP1R3B*‐mediated metabolic pathways may therefore represent a promising therapeutic strategy for modulating macrophage function and mitigating atherosclerotic progression. Moreover, *PPP1R3B* emerges as a metabolic checkpoint linking oxygen availability to substrate utilization and macrophage polarization. Its reversible suppression upon reoxygenation underscores its potential as a dynamic regulator of immune‐metabolic balance. Together, these findings establish a mechanistic link between local hypoxia, metabolic dysfunction, and macrophage maladaptation, offering new targets for metabolic reprogramming in advanced atherosclerosis.

### 2.3 *PPP1R3B* Induces Anti‐AS by Reprogramming Glycogen Metabolism to Promote M2 MΦ Polarization

**Figure 3:**
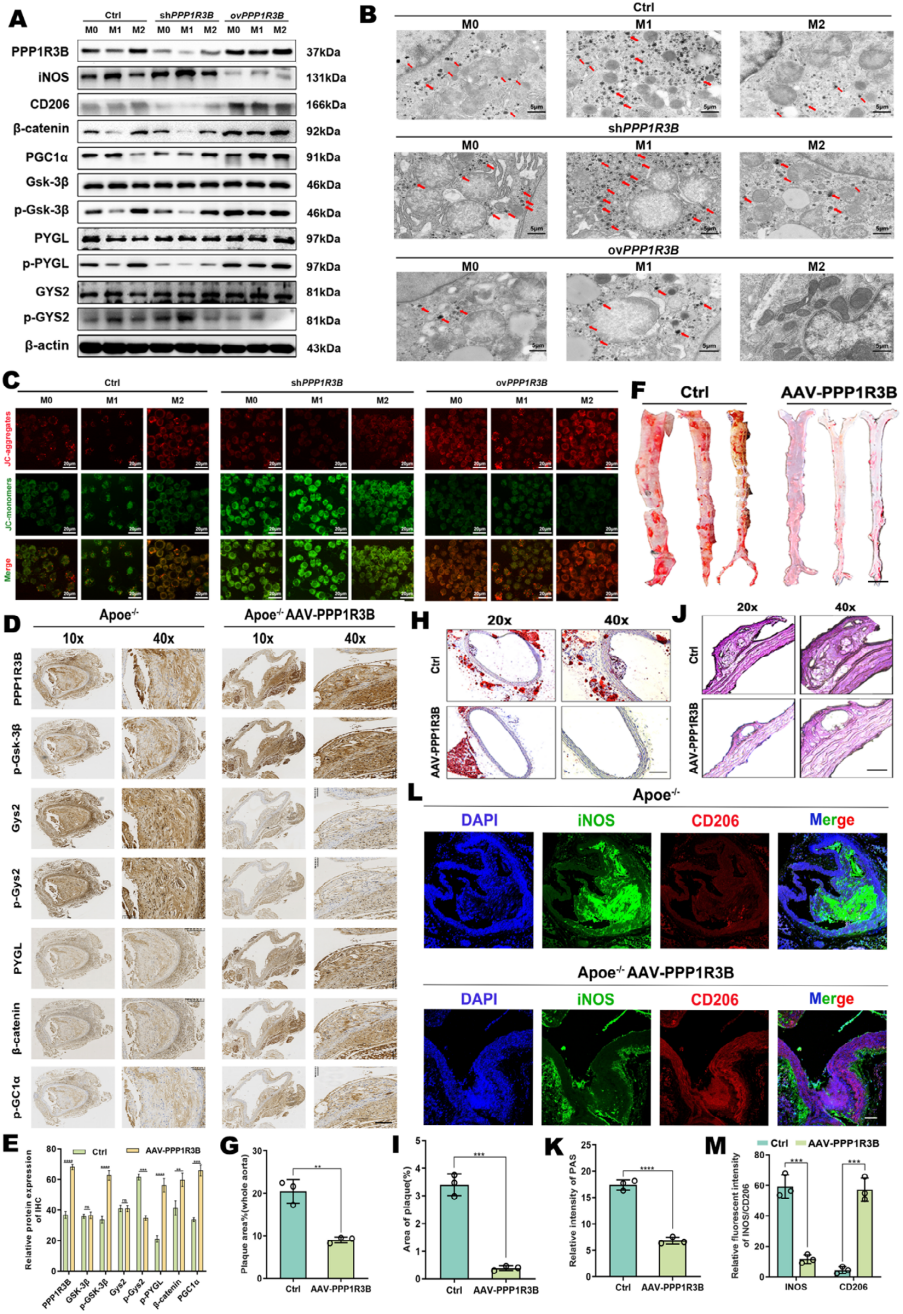
*PPP1R3B* inhibits the deterioration of atherosclerotic plaques by regulating the glycogen metabolism reprogramming of M2 MΦs in vitro and in vivo. A) Western blot following the overexpression (ov*PPP1R3B*) or knockdown (sh*PPP1R3B*) of *PPP1R3B* in M0, M1, and M2 MΦs. B) Representative TEM images of glycogen granules in M0, M1, and M2 MΦs from the different treatment groups. Scale bar: 5 µm. C) Representative fluorescence images of JC‐1 staining in M0, M1, and M2 MΦs transfected with ov*PPP1R3B* and sh*PPP1R3B* (red = aggregates, green = monomers). Scale bar: 20 µm. D) Representative images of IHC staining in *Apoe* ^−/−^ mice with or without transfection with AAV‐*PPP1R3B*, an AAV vector that overexpresses *PPP1R3B*. Scale bar: 40 µm. E) Quantized statistical histogram of (D). F) Representative images of ORO staining in the aorta of the control and AAV‐*PPP1R3B* groups. Scale bar: 40 µm. G) Quantized statistical histogram of (F). H) Representative images of ORO staining in aortic cross‐sections of the control and AAV‐*PPP1R3B* groups. Scale bar: 40 µm. I) Quantized statistical histogram of (H). J) Representative images of PAS staining in aortic cross‐sections of the control and AAV‐*PPP1R3B* groups. Scale bar: 40 µm. K) Quantized statistical histogram of (J). L) Representative immunofluorescence images of 4’,6‐diamidino‐2‐phenylindole (DAPI; blue), iNOS (green), and CD206 (red) staining in aortic root tissue sections of *Apoe* ^−/−^ mice from the control and AAV‐*PPP1R3B* groups. Scale bar: 20 µm. M) Quantized statistical histogram of (L). The data are presented as the mean ± SEM across three biologically independent samples. Significance: *p < 0.05; **p < 0.01; ***p < 0.001; ****p < 0.0001.

In vivo MΦs *PPP1R3B* transgenic AS mouse models were established to explore the regulatory role of *PPP1R3B* on the immunometabolism of MΦs associated with AS. In these models, overexpressing *PPP1R3B* significantly promoted the expression of key proteins involved in the glycogen degradation metabolic signaling pathway (e.g., phosphorylated [p]‐glycogen synthase kinase 3 beta [GSK3B/GSK‐3β] and p‐glycogen phosphorylase L [PYGL]) and the M2 MΦ polarization signaling pathway (p‐GSK‐3β, catenin beta 1 [CTNNB1] and PPARG coactivator 1 alpha [PPARGC1A/PGC‐1α], and CD206). In contrast, overexpressing *PPP1R3B* markedly inhibited the expression of key proteins in the glycogen synthesis metabolic signaling pathway, including p‐glycogen synthase 2 [GYS2]) and the M1 MΦ polarization signaling pathway (iNOS; **Figure** **3**A). Transmission electron microscopy (TEM) revealed that, compared to the Ctrl group, glycogen granules (indicated by red arrows) accumulated significantly in sh*PPP1R3B*‐M1 MΦs, while their levels were markedly reduced in ov*PPP1R3B*‐M2 MΦs (Figure 3B). This observation suggests that overexpression of *PPP1R3B* effectively promotes glycogen degradation in M2 MΦs, whereas its knockdown facilitates glycogen accumulation in M1 MΦs. This differential regulatory effect may be attributed to the precise modulation of key enzymes involved in glycogen metabolism, underscoring its critical role in metabolic reprogramming within MΦ polarization. Next, the influence of *PPP1R3B* on mitochondrial function in MΦs was examined using a JC‐1 mitochondrial membrane potential assay. M2 MΦ polarization and *PPP1R3B* overexpression groups showed enhanced JC‐1 aggregate fluorescence over Ctrl groups, which revealed that overexpressing *PPP1R3B* elevated the mitochondrial membrane potential in M2 MΦs. Conversely, knocking down *PPP1R3B* decreased the mitochondrial membrane potential in M1 MΦs, as evidenced by green fluorescent JC‐monomers (Figure 3C; Figure S10A, Supporting Information).

To further clarify the immunometabolic regulatory role of *PPP1R3B* in AS‐related MΦs in vivo, an AAV‐mediated overexpression model of *PPP1R3B* in *Apoe* ^−/−^ mice (denoted as *Apoe* ^−/−^AAV‐*PPP1R3B*) was successfully established (Figure S10B,C, Supporting Information). In the *Apoe* ^−/−^mouse, overexpression of *PPP1R3B* significantly modulates key signaling pathways involved in glycogen metabolism and MΦ polarization. Specifically, upregulation of *PPP1R3B* resulted in a notable increase in the expression of critical proteins associated with glycogen degradation, such as p‐GSK‐3β and p‐PYGL. Key molecules in the M2 MΦ polarization pathway were also significantly upregulated, including p‐GSK‐3β, CTNNB1, and p‐PGC‐1α. In contrast, the expression of p‐GYS2, a crucial protein in the glycogen synthesis pathway, was remarkably decreased (Figure 3D,E; Figure S10D, E, Supporting Information). Consistent with the Oil Red O staining results demonstrating reduced plaque formation in *PPP1R3B*‐overexpressing aortae (Figure 3F,G), cross‐sectional H&E analysis further corroborated a significant decrease in plaque area within the vascular lumen of the overexpression group (Figure 3H,I). Not only *PPP1R3B* overexpression enhances glycogen degradation and M2 MΦ polarization signaling, but also suppresses glycogen synthesis, thereby regulating both metabolic and immune microenvironments and inhibiting the formation of atherosclerotic plaques and glycogen accumulation (Figure 3J,K; Figure S10F,G, Supporting Information). Meanwhile, M1 polarization of MΦs within plaques was inhibited, contributing to the suppression of atherosclerotic plaque formation (Figure 3L,M; Figure S10H,I, Supporting Information). In conclusion, modulating *PPP1R3B* expression demonstrated that *PPP1R3B* can trigger the M2 polarization of MΦs by regulating MΦ glycogen catabolism for treatment of AS (Figure S10J, Supporting Information).

### 2.4 STAT3 is Crucial in the Regulation of MΦs Immune Metabolism by *PPP1R3B*

**Figure 4:**
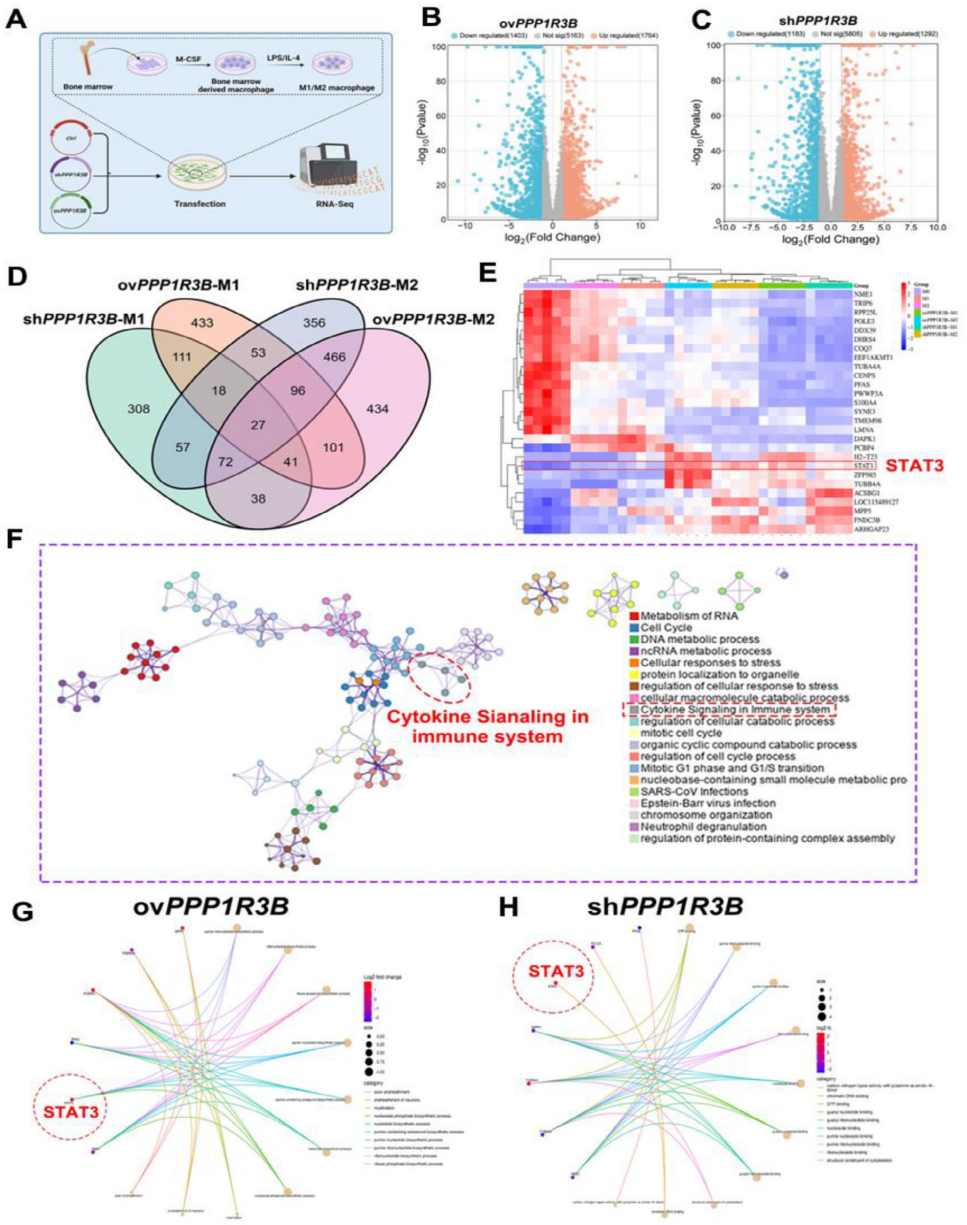
Transcriptome analysis of *PPP1R3B*’s regulation of AS‐related MΦ immunometabolism via STAT3. A) Scheme of cell transfection and RNA‐seq analysis of sh*PPP1R3B* and ov*PPP1R3B* in various MΦ types. Volcano maps of RNA‐seq data for MΦs treated with B) ov*PPP1R3B* and C) sh*PPP1R3B*. D) Quadruple Venn diagram analysis of DEGs in M1/M2 MΦs treated with ov*PPP1R3B* and sh*PPP1R3B*. E) Heatmap of DEGs in M1/M2 MΦs treated with ov*PPP1R3B* and sh*PPP1R3B*. F) Metascape analysis of DEGs in M1/M2 MΦs treated with ov*PPP1R3B* and sh*PPP1R3B*. Network maps of the functional enrichment analysis of DEGs in M1/M2 MΦs treated with G) ov*PP1R3B* and H) sh*PPP1R3B*. The data are presented across three biologically independent samples.

RNA‐seq was used to examine various MΦ types within the ov*PPP1R3B* and sh*PPP1R3B* groups to extensively investigate the immunometabolic targets of *PPP1R3B*‐regulated AS‐related MΦs (**Figure** **4**A). Overexpressing *PPP1R3B* in M1 and M2 MΦs upregulated 1764 and downregulated 1403 genes (Figure 4B). Conversely, knocking down *PPP1R3B* in M1 and M2 MΦs upregulated 1292 and downregulated 1183 genes (Figure 4C). The DEGs in the ov*PPP1R3B* and sh*PPP1R3B* MΦs groups were examined using weighted gene co‐expression network analysis (WGCNA), and the most significant module characteristic genes (|*r*| > 0.9, *p* < 0.05) were identified (Figures S11, S12A–D, Supporting Information). The characteristic genes in the “dark orange” module (|*r*| = 0.99, *p* < 0.05) in ov*PPP1R3B*‐M1, “turquoise” module (|*r*| = 0.99, *p* < 0.05) in ov*PPP1R3B*‐M2, “blue” module (|*r*| = 0.96, *p* < 0.05) in sh*PPP1R3B*‐M1, and “turquois”’ module (|*r*| = 0.99, *p* < 0.05) in sh*PPP1R3B*‐M1 were selected for further analysis.

Next, the DEGs in M1/M2 MΦs in the ov*PPP1R3B* and sh*PPP1R3B* groups were analyzed using WGCNA and visualized in Venn diagrams and heatmaps (Figure 4D,E).

The analysis identified 27 differentially expressed genes (DEGs) involved in the regulation of MΦ immune metabolism by *PPP1R3B*, providing insight into the complex gene interactions influencing this process. Metascape, GSEA, and GO were used to examine the regulatory effects of *PPP1R3B* on the 27 DEGs related to the immune metabolism of MΦs (Figure 4F; Figure S12E–H, Supporting Information). *PPP1R3B* was found to regulate the expression of these DEGs mainly through nucleotide biosynthesis (e.g., purine ribonucleotide biosynthesis, purine nucleotide biosynthesis, and purine nucleotide biosynthesis), the biosynthesis of key mitochondrial components (e.g., ubiquinone), and the cytokine signal transduction pathway, which further affects the metabolic processes of MΦs (e.g., lipid and purine metabolism). Interestingly, the network map from the gene functional enrichment analysis further revealed that STAT3 was a key link in the MΦs immune metabolism signaling pathway regulated by *PPP1R3B* (Figure 4G,H). IP‐MS analysis identified STAT3 as a high‐confidence interacting partner of PPP1R3B. Although several glycogen metabolism‐related enzymes, such as GYS1 and PYGL, were also detected, their enrichment signals were relatively weak and failed to be validated in subsequent assays. Due to its strong interaction and well‐established role in immunometabolic regulation, STAT3 was selected as the primary focus for downstream functional investigations (Table S2, Supporting Information).

### 2.5 *PPP1R3B* Facilitates M2 MΦ Polarization and Enhances Mitochondrial Glycogenolysis via STAT3 in Vitro

**Figure 5:**
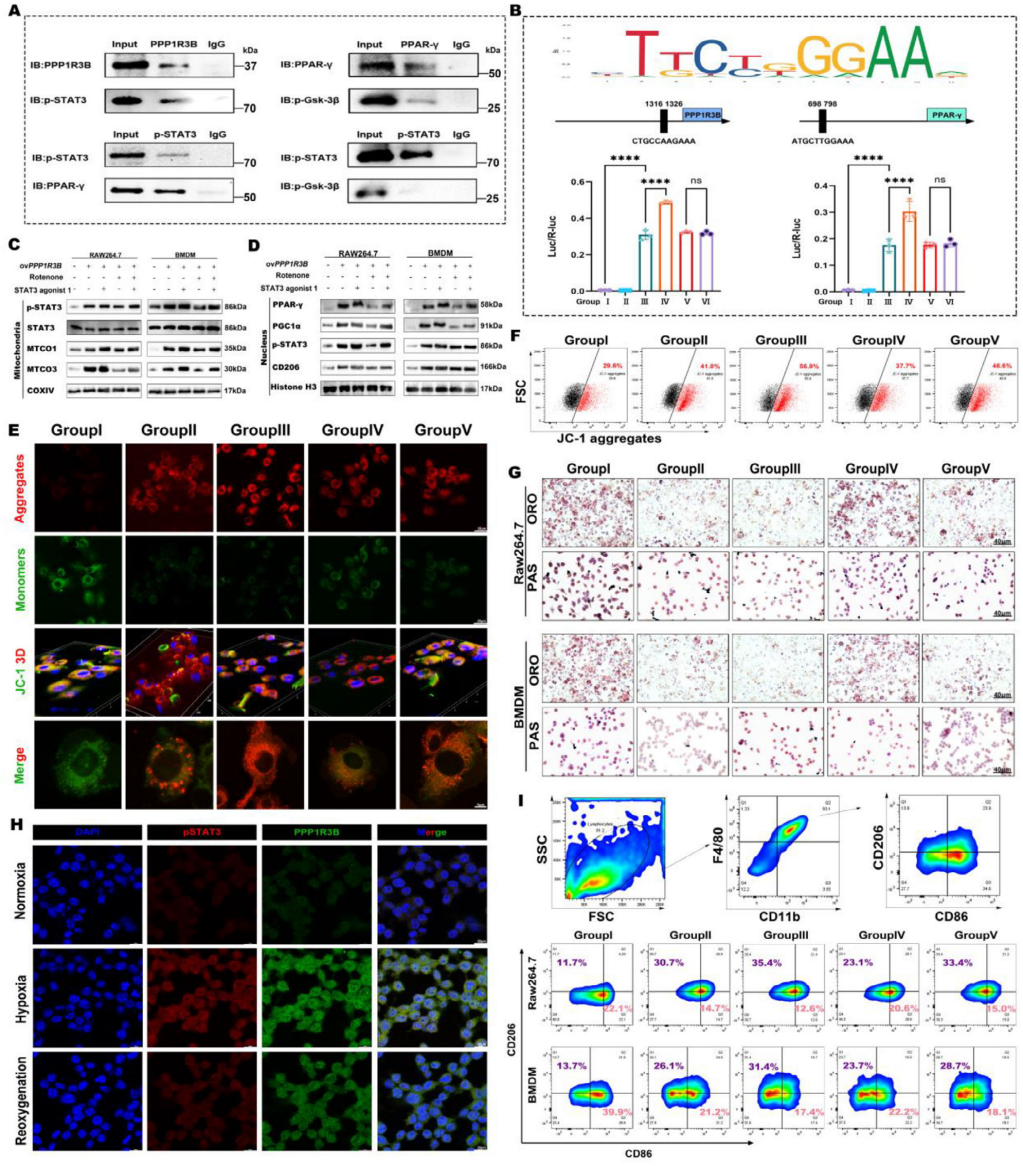
*PPP1R3B* promotes glycogen metabolism reprogramming and M2 MΦ polarization by activating the STAT3 signal pathway. A) Co‐IP analysis of the interactions among *PPP1R3B*, PPAR‐γ, p‐GSK‐3β, p‐STAT3. B) Dual‐luciferase reporter analysis of STAT3 regulating *PPP1R3B* and *PPAR‐γ* transcription. Group I: *PGL4.10‐NC+pcDNA3.1(+)‐3xFLAG‐P2A‐EGFP*, Group II: *PGL4.10‐NC+pcDNA3.1(+)‐STAT3‐3xFLAG‐P2A‐EGFP*, Group III: *PGL4.10‐PPAR‐γ promoter (WT)+pcDNA3.1(+)‐3xFLAG‐P2A‐EGFP*, Group IV: *PGL4.10‐PPAR‐γ promoter (WT)+pcDNA3.1(+)‐STAT3‐3xFLAG‐P2A‐EGFP*, Group V: *PGL4.10‐PPAR‐γ promoter (MUT)+pcDNA3.1(+)‐3xFLAG‐P2A‐EGFP*, Group VI: *PGL4.10‐PPAR‐γ promoter (MUT)+pcDNA3.1(+)‐STAT3‐3xFLAG‐P2A‐EGFP*. Western blots of C) mitochondrial and D) nuclear proteins in MΦs (Raw264.7 and BMDM) in the different treatment groups. E) Representative JC‐1 fluorescence staining images and F) flow cytometry analysis of BMDMs in the different treatment groups. G) Representative images of ORO and PAS staining in MΦs (RAW264.7 and BMDM) in the different treatment groups. H) Representative immunofluorescence images depict PPP1R3B (green) and pSTAT3 (red) expression in M2 macrophages under normoxia, hypoxia, and reoxygenation. Scale bar: 20 µm. I) Flow cytometry analysis of M1 (F4/80^+^ and CD86^+^) and M2 (F4/80^+^ and CD206^+^) MΦs (RAW264.7 and BMDM) in the different treatment groups. The data are represented as the mean ± SEM across three biologically independent samples. Significance: *p < 0.05; **p < 0.01; ***p < 0.001; ****p < 0.0001. Treatment groups: I, control; II, ov*PPP1R3B*; III, ov*PPP1R3B*+STAT3 agonist 1; IV, ov*PPP1R3B*+rotenone; V, ov*PPP1R3B*+STAT3 agonist 1+rotenone. The data are presented across three biologically independent samples. Scale bar: represent 20 µm.

The intricacies of the *PPP1R3B* signaling pathway were examined in vitro to elucidate its regulatory role in the immune metabolism of atherosclerotic MΦs via STAT3. The proteins interacting with *PPP1R3B* were identified by immunoprecipitation (IP) and mass spectrometry (MS), which revealed that *PPP1R3B* can bind to STAT3 and other inflammatory immunometabolic cytokine‐related proteins (Figure S13A, Supporting Information). Given the combined IP and MS data above, the interactions of *PPP1R3B* with proteins related to the immune metabolism of MΦs were further explored by co‐IP. *PPP1R3B* was demonstrated to directly bind phosphorylated STAT3 (p‐STAT3), which, in turn, directly interacts with PPAR‐γ. Additionally, PPAR‐γ was shown to bind directly to phosphorylated GSK‐3β (p‐GSK‐3β) (**Figure** **5**A). However, no direct interaction was observed between p‐STAT3 and p‐GSK‐3β. Bioinformatic analysis predicted STAT3 binding sites within the promoter regions of *PPP1R3B* and PPAR‐γ. They were identified at −1326 and −1316 bp in the *PPP1R3B* promoter and at −798 and −698 bp in the *PPAR‐γ* promoter related to the transcription initiation site. Additionally, dual‐luciferase reporter assays revealed that STAT3 increased *PPP1R3B* and *PPAR‐γ* promotor‐driven luciferase activity (Figure 5B).

To further elucidate how the *PPP1R3B*/STAT3 signaling pathway regulates immune metabolism in MΦs, two MΦ types (Raw 264.7 cells and BMDMs) were divided into five distinct treatment groups: Group I, control; Group II, ov*PPP1R3B*; Group III, ov*PPP1R3B*+STAT3 agonist 1 (α7 nAchR‐JAK2‐STAT3 agonist 1); Group IV, ov*PPP1R3B*+rotenone; and Group V, ov*PPP1R3B*+STAT3 agonist 1+rotenone. After treatment, mitochondrial and nuclear proteins were extracted from each group and examined by western blotting. The results demonstrated that treating MΦs with ov*PPP1R3B* and 20 µM STAT3 agonist 1 (Group III) increased the levels of *PPP1R3B*, p‐STAT3, and mitochondrial activity markers such as mitochondrial cytochrome c oxidase subunits I (MT‐CO1) and III (MT‐CO3). However, treating MΦs with 2.5 µM rotenone (Groups IV and V) also inhibited these effects (Figure 5C). In addition, western blot analysis of the nuclear proteins revealed that increasing the expression of *PPP1R3B* and STAT3 in MΦs (Group III) significantly increased the levels of PPAR‐γ, PGC‐1α, p‐STAT3, and CD206, facilitating their M2 polarization (Figure 5D).

To comprehensively explore the regulatory effect of the *PPP1R3B*/STAT3 signaling pathway on the immunometabolic functions of MΦs, post‐treatment mitochondrial function, glycogen metabolism, cholesterol metabolism, and polarization phenotype in MΦs were further investigated in the above five treatment groups. The JC‐1 mitochondrial membrane potential assay showed overexpressing *PPP1R3B* effectively increased mitochondrial activity in BMDMs, and 20 µM STAT3 agonist 1 further increased *PPP1R3B*‐mediated mitochondrial activation (Group III). However, also treating MΦs with 2.5 µM rotenone inhibited this enhanced mitochondrial activation (Figure 5E,F; Figure S13B,C, Supporting Information). In addition, combining *PPP1R3B* overexpression with STAT3 activation significantly enhanced glycogen degradation in MΦs and cholesterol excretion by MΦ‐derived foam cells (Group III). However, blocking mitochondrial activity with 2.5 µM rotenone (Groups IV and V) reduced lipid and glycogen degradation excretion in MΦs (Figure 5G; Figure S13D,E, Supporting Information).

To further elucidate the spatiotemporal regulatory mechanisms of PPP1R3B expression under hypoxic stress, we investigated STAT3 activation in M2 macrophages across varying oxygenation states. Immunofluorescence analysis revealed that under hypoxia, both phosphorylated STAT3 (p‐STAT3) and *PPP1R3B* were markedly upregulated and co‐localized in the cytoplasm and perinuclear regions, suggesting coordinated activation and potential functional interplay. Upon reoxygenation, the fluorescence intensity of both molecules significantly decreased, indicating that their expression is dynamically regulated in an oxygen‐dependent and reversible manner (Figure 5H; Figure S13F, Supporting Information). Notably, the activation of p‐STAT3 temporally preceded and spatially coincided with *PPP1R3B* induction, supporting the hypothesis that p‐STAT3 may act upstream in driving *PPP1R3B* transcription and subcellular localization. Together, these findings suggest that the p‐STAT3/PPP1R3B axis constitutes a critical oxygen‐sensitive signaling module, enabling M2 macrophages to adapt metabolically to fluctuating oxygen levels within the atherosclerotic plaque microenvironment.

Interestingly, flow cytometry revealed that overexpressing *PPP1R3B* and activating STAT3 in MΦs (Group III) led to M2 polarization while blocking mitochondrial function (Groups IV and V) contributed to M1 polarization (Figure 5I; Figure S13G,H, Supporting Information). Based on the results above, it is evident that *PPP1R3B* plays a role in the STAT3‐mediated signaling cascade, contributing to the enhancement of mitochondrial activity.

To confirm the direct interaction between PPP1R3B and phosphorylated STAT3 (p‐STAT3), a GST pull‐down assay was performed. The results demonstrated a specific binding between PPP1R3B and endogenously phosphorylated STAT3, further supporting the post‐translational regulatory axis identified by IP‐MS and co‐IP (Figure S14, Supporting Information). To further validate the functional role of p‐STAT3 in PPP1R3B‐mediated immunometabolic remodeling, a STAT3‐specific inhibitor (Stattic) was employed. Western blot analysis revealed a dose‐dependent inhibition of glycogen degradation markers and M2 MΦ polarization markers upon Stattic treatment (Figure S15A, Supporting Information). Consistently, mitochondrial membrane potential was reduced in a concentration‐dependent manner, as indicated by JC‐1 fluorescence shift (Figure S15B,C, Supporting Information). In parallel, PAS staining confirmed a marked increase in intracellular glycogen accumulation following p‐STAT3 inhibition (Figure S15D,E, Supporting Information), highlighting the essential role of p‐STAT3 in mediating mitochondrial glycogenolysis.

In summary, these data suggest that *PPP1R3B* binds to phosphorylates STAT3, and activates it. *PPP1R3B*‐activated p‐STAT3 promotes the expression of *MT‐CO1* and *MT‐CO3*, pivotal components of mitochondrial energy metabolism, thereby enhancing the degradation and energy supply derived from glycogen and lipids within mitochondria. It also enhances M2 MΦ polarization by increasing the expression of the key polarization‐induced nuclear proteins PPAR‐γ, PGC‐1α, and CD206 (Figure S16, Supporting Information).

While *PPP1R3B* is well‐recognized as a key regulator of glycogen metabolism, this study significantly extends its known functions by revealing its unique role in modulating immunometabolic signaling within atherosclerotic plaques. Unlike prior research, which primarily emphasized its role in maintaining metabolic homeostasis in systemic tissues such as the liver and muscles, this work is the first to establish *PPP1R3B* as a critical mediator of MΦ polarization and metabolic reprogramming under hypoxic and inflammatory conditions.

### 2.6 *PPP1R3B* Induces M2 MΦ Polarization and Glycogen Metabolic Reprogramming via the STAT3 Signaling Pathway to Treat AS in Vivo

**Figure 6:**
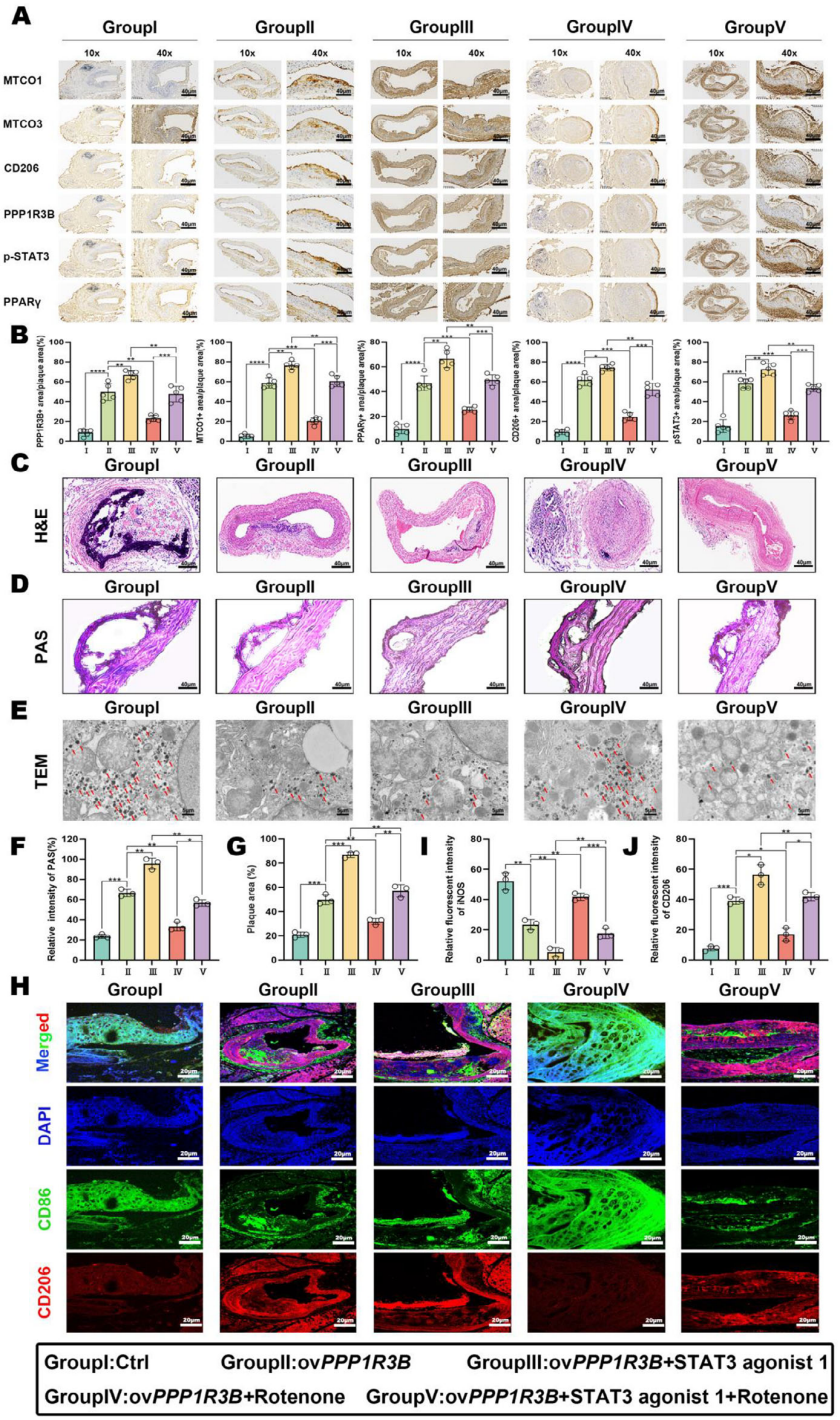
Upregulation of *PPP1R3B* in vivo inhibits AS by activating STAT3 signaling pathway‐induced glycogen metabolic reprogramming and M2 MΦs. A) Representative images of IHC staining of cross‐sections of aortic tissue in each treatment group. Scale bar: 40 µm. B) Quantized statistical histogram of (A). Representative images of C) hematoxylin and eosin and D) PAS staining of cross‐sections of aortic tissue from each treatment group. Scale bar: 40 µm. E) Representative TEM images of glycogen granules in *Apoe* ^−/−^ mice in the different treatment groups. Scale bar: 5 µm. F) Quantized statistical histogram of (C). G) Quantized statistical histogram of (D). H) Representative immunofluorescence images of DAPI (blue), CD86 (green), and CD206 (red) staining in cross‐sections of aortic tissue from each treatment group. Scale bar: 20 µm. The data are presented as the mean ± SEM across three biologically independent samples. Significance: *p < 0.05; **p < 0.01; ***p < 0.001; ****p < 0.0001. Treatment groups: I, control; II, ov*PPP1R3B*; III, ov*PPP1R3B*+STAT3 agonist 1; IV, ov*PPP1R3B*+rotenone; V, ov*PPP1R3B*+STAT3 agonist 1+rotenone.

To further explore the anti‐atherosclerotic mechanism of the *PPP1R3B*/STAT3 signal pathway in vivo, 25 *Apoe* ^−/−^ mice were randomly divided into the following five groups: Group I, control; Group II, ov*PPP1R3B*; Group III, ov*PPP1R3B*+STAT3 agonist 1 (1 mg kg^−1^ in 20% DMSO); Group IV, ov*PPP1R3B*+rotenone (2 mg kg^−1^ in 20% DMSO); and Group V, ov*PPP1R3B*+STAT3 agonist 1 (1 mg kg^−1^ in 20% DMSO)+rotenone (2 mg kg^−1^ in 20% DMSO). Immunohistochemical (IHC) staining and western blotting of the aortic plaques of *Apoe* ^−/−^ mice in these five treatment groups confirmed that overexpressing *PPP1R3B* activated p‐STAT3 (Group III) significantly enhanced the expression of key proteins involved in mitochondrial activation (e.g., MT‐CO1 and MT‐CO3) and M2 MΦ polarization (e.g., PGC‐1α, CD206, and PPAR‐γ). In contrast, blocking mitochondrial activity with rotenone (Groups IV and V) markedly inhibited these effects (**Figure** **6**A,B; Figure S17A,B, Supporting Information).

In addition, activating the *PPP1R3B*/STAT3 signaling pathway significantly enhanced the regression of atherosclerotic plaques and stimulated the degradation of glycogen in vivo. Notably, combining *PPP1R3B* overexpression with p‐STAT3 activation (Group III) significantly decreased the atherosclerotic plaque area by 27% and increased the glycogen catabolic rate to 86.7% compared to Group I (Figure 6C–G). However, blocking the mitochondrial electron transport chain (ETC) with rotenone (Groups IV and V) significantly inhibited the combined anti‐atherosclerotic effects of *PPP1R3B* and p‐STAT3. Next, to further clarify the mechanism of *PPP1R3B*/STAT3 signal pathway to regulate the inflammatory immune microenvironment in atherosclerotic plaques, immunofluorescence staining and flow cytometry were employed to examine those from *Apoe* ^−/−^ mice in the above five treatment groups (Figure 6H; Figure S17C–E, Supporting Information). These results showed that activating the *PPP1R3B*/STAT3 signaling pathway led to significant remodeling of the inflammatory immune microenvironment in plaques, resulting in a significant enrichment of CD206^+^ anti‐inflammatory M2 MΦs and forkhead box P3 (FOXP3) ^+^ regulatory T cells (Tregs) in plaques.

Our results indicate that *PPP1R3B* regulates STAT3 signaling via a dual mechanism. At the transcriptional level, *PPP1R3B* modestly increases STAT3 mRNA expression (Figure 4), though this alone does not fully account for downstream effects. More importantly, IP‐MS and co‐IP assays confirmed a direct interaction between *PPP1R3B* and phosphorylated STAT3 (p‐STAT3) (Figure 5), which is essential for *PPP1R3B*‐driven glycogen metabolic remodeling and M2 MΦ polarization, as shown by Stattic inhibition experiments (Figure S15, Supporting Information). Collectively, these findings support a two‐tiered model in which *PPP1R3B* modulates both the expression and activation of STAT3, enabling precise control of immunometabolic processes in atherosclerosis.

## 3 Discussion

This study employed high‐throughput RNA sequencing (RNA‐seq) and bioinformatics approaches to investigate the immunometabolic regulatory network of MΦs within human atherosclerotic plaques. The goal was to enhance the adaptability and remodeling capacity of anti‐inflammatory and reparative M2 MΦs in the complex inflammatory metabolic microenvironment of atherosclerotic plaques. *PPP1R3B* is a key immunometabolic target to promote the M2 polarization of MΦs. Further analysis revealed that the absence of *PPP1R3B* in M2 MΦs correlated significantly with the exacerbation of human atherosclerotic plaques, underscoring the pivotal role of *PPP1R3B* in modulating ASCVD pathogenesis (Figure 1).

*PPP1R3B* regulates glycogen synthesis and breakdown as a regulatory subunit of the PP1 complex. It facilitates the binding of the PP1 catalytic subunit to glycogen granules, aiding in the process of glycogen metabolism.^[^ ^22^, ^25^, ^28^ ^]^ *PPP1R3B*, a key regulator of glycogen metabolism, is a critical metabolic regulatory site intricately linked to the risk of developing ASCVD.^[^ ^27^, ^29^, ^30^ ^]^ In glycogen metabolism, *PPP1R3B* influences glycogen synthesis by modulating the dephosphorylation of glycogen synthase and glycogen phosphatase via PP1.^[^ ^25^ ^]^ Dephosphorylation activates glycogen synthase, increasing glycogen synthesis, but inactivates glycogen phosphatase, decreasing glycogen breakdown.^[^ ^22^ ^]^

Glycogen, a key polysaccharide energy reserve, is predominantly stored in the liver and muscles.^[^ ^22^ ^]^ Traditionally, glycogen accumulation in the liver is thought to contribute to metabolic syndromes, such as ASCVD.^[^ ^23^ ^]^ Enhancing *PPP1R3B* expression not only promotes glycogen storage in the liver but also significantly reduces serum LDL‐C levels and the risk of myocardial infarction.^[^ ^23^, ^25^ ^]^ Furthermore, high *PPP1R3B* expression has been associated with improved lipid profiles—including levels of high‐density lipoprotein‐cholesterol, LDL‐C, and total cholesterol—and significantly reduced plasma inflammatory markers and neutrophil counts^[^ ^23^, ^25^, ^27^, ^31^ ^]^ While these findings emphasize the potential role of *PPP1R3B* in regulating immune metabolism linked to ASCVD, further detailed studies are required to elucidate how it influences this regulatory process.

This study concentrated on the role of *PPP1R3B* in modulating MΦs metabolism, a key immune component in AS, to understand the immunometabolic regulatory mechanisms of *PPP1R3B* in treating ASCVD. Overexpressing *PPP1R3B* significantly enhances the M2 MΦ polarization, mitochondrial activity, lipid excretion (e.g., cholesterol), and glycogen degradation of MΦs. Conversely, downregulating *PPP1R3B* has the opposite effect. overexpressing *PPP1R3B* in both in vivo and in vitro atherosclerotic models not only activated the glycogen degradation regulatory axis (p‐GSK‐3β/p‐PYGL) to promote glycogen degradation but also stimulated the M2 polarization regulatory axis in plaque MΦs (p‐GSK‐3β/CTNNB1/p‐PGC‐1α/CD206), thereby enhancing the M2 polarization of MΦs within plaques. Based on these findings, we hypothesize that *PPP1R3B*‐mediated metabolic reprogramming of glycogen degradation not only provides essential energy substrates for M2 MΦ polarization but also enhances their ability to efficiently utilize these substrates through improved mitochondrial activity. Moreover, extensive studies have demonstrated that the polarization of M2 MΦs predominantly depends on glucose‐fueled OXPHOS, with fatty acid oxidation (FAO) acting as an auxiliary ATP source under conditions of glucose deprivation.^[^ ^32^, ^33^, ^34^, ^35^ ^]^

Suppressing glycolysis can also downregulate the expression of phenotypic genes in M2 MΦs.^[^ ^21^ ^]^ The degradation of glycogen, a vital energy reserve, creates glucose and its phosphorylated derivatives, generating substantial ATP via the glycolysis and OXPHOS pathways.^[^ ^36^ ^]^ Significantly, overexpression of *PPP1R3B* effectively increased glycolysis end products while reducing FAO.^[^ ^23^, ^37^ ^]^ Notably, glucocorticoids also contributes to significantly upregulate *PPP1R3B* expression. Therefore, *PPP1R3B*‐mediated reprogramming of glycogen degradation facilitates the M2 polarization of MΦs and the treatment of atherosclerotic plaques by providing the energy substrate glucose and enhancing mitochondrial activity.

Next, this study sought to clarify how *PPP1R3B* simultaneously enhances the signaling pathways involved in M2 MΦ polarization and the mitochondrial reuse of energy from glycogen degradation products. It utilized high‐throughput RNA‐seq, co‐IP, and dual‐luciferase reporter assays to demonstrate that *PPP1R3B* directly binds to phosphorylates STAT3 and activates it. Further investigation revealed that activated p‐STAT3 translocates into the nucleus, where it initiates the transcriptional activity of *PPAR‐γ* and key signaling pathways involved in M2 MΦ polarization, including the expression of *STAT3*, *PPAR‐γ*, *PGC‐1α*, and *CD206* (Figure 5). While our data support the existence of both nuclear and mitochondrial arms of the STAT3 signaling axis, the detailed spatiotemporal localization patterns and chromatin‐binding characteristics remain to be fully elucidated in future studies. Nevertheless, these findings strongly suggest that *PPP1R3B* regulates a dual‐compartment STAT3 pathway, enabling both transcriptional programming and metabolic activation that collectively promote anti‐inflammatory M2 MΦ phenotypes in atherosclerotic lesions.

Moreover, activated p‐STAT3 enters mitochondria, enhancing their membrane potential and respiratory chain activity and activating the expression of mitochondrial proteins *MT‐CO1* and *MT‐CO3*, promoting glycogen degradation (Figure 5). Numerous studies have demonstrated that phosphorylation at Ser727 causes STAT3 to migrate to the mitochondria, inducing FAO and OXPHOS to enhance ATP production.^[^ ^38^, ^39^, ^40^ ^]^ This phosphorylated form of STAT3 (Ser727) is particularly crucial since it interacts primarily with mitochondrial ETC complexes I and III, encoded by MT‐CO1 and MT‐CO3, respectively.^[^ ^38^, ^40^ ^]^ This interaction is a pivotal trigger for activating mitochondrial OXPHOS, enhancing the energy supply required for the anti‐inflammatory actions of M2 MΦs, thereby contributing to treating AS.^[^ ^40^, ^41^ ^]^ Furthermore, other studies have confirmed that p‐STAT3 (Ser727) also plays a significant role in immune cells under hypoxic conditions by activating the glycogen metabolic signaling pathway GYS1/glycogen phosphorylase B (PYGB), which accelerates the rapid utilization and energy supply from glycogen catabolism in mitochondria.^[^ ^41^, ^42^ ^]^

Although our research has elucidated the functional role of *PPP1R3B* in MΦ metabolism and inflammation using preclinical models, we recognize that translating these findings to humans may present significant challenges. These challenges include tissue‐specific functions of *PPP1R3B* and the potential for off‐target effects due to systemic regulation. Future studies should prioritize the development of tissue or cell type‐specific delivery systems, such as nanoparticle platforms that selectively target diseased MΦs, or the use of conditional knockout models to further delineate environment‐specific effects. Concerning cell type specificity, while our transcriptomic and functional data suggest that *PPP1R3B* predominantly regulates immune‐metabolic crosstalk in MΦs, additional analyses are required to comprehensively evaluate its regulatory role, particularly under inflammatory conditions. Nonetheless, further investigation is warranted to fully assess and exclude any potential roles of *PPP1R3B* in non‐MΦ populations, especially in advanced atherosclerosis or metabolic disorders. To address these translational barriers, future studies should prioritize the development of targeted delivery systems that enable cell type–specific modulation of *PPP1R3B*. Promising strategies include lipid‐based nanoparticles, aptamer‐conjugated carriers, or other biomaterial platforms engineered to selectively target lesional macrophages. In parallel, conditional genetic models (e.g., LysM‐Cre or CX3CR1‐Cre–driven *PPP1R3B* knockout or overexpression) can be employed to clarify the immune‐specific functions of *PPP1R3B* in vivo. Ultimately, successful translation will require not only mechanistic dissection in relevant disease models but also validation in large animals and the integration of precision delivery approaches to ensure both efficacy and safety in clinical applications. This study consistently found that in both in vivo and in vitro atherosclerotic models, overexpressing *PPP1R3B* in MΦs and treating them with the mitochondrial activator STAT3 agonist 1 significantly enhanced their mitochondrial activity and glycogen degradation capabilities, facilitating their M2 polarization. However, these effects were significantly reduced by the mitochondrial blocker rotenone (Figures 5 and 6). Collectively, these results indicate that *PPP1R3B*‐activated STAT3 is crucial in driving the M2 polarization of MΦs and orchestrating programmed glycogen degradation in mitochondria, offering potential therapeutic targets in treating AS.

## 4 Conclusion

In summary, overexpression of *PPP1R3B* in plaque MΦs activates the p‐STAT3/PPAR‐γ/PGC‐1α/CD206 axis, and induces the M2 polarization of MΦs, which can prevent the hypoxic plaque environment from interfering with their immunometabolism. It also simultaneously activates the p‐STAT3/p‐GSK‐3β/p‐PYGL/p‐GYS2 axis in M2 MΦs, enhancing their mitochondrial metabolism and energy supply from glycogen particles, thereby inducing them to excrete cholesterol into the plaques and resist ASCVD.

## Conflict of Interest

The authors declare no conflict of interest.

## Supporting information



Supporting Information

Supplementary Table 1

Supplementary Table 2

Supplementary Table 3

## Data Availability

The data that support the findings of this study are available on request from the corresponding author. The data are not publicly available due to privacy or ethical restrictions.

## References

[1] C. W. Tsao , A. W. Aday , Z. I. Almarzooq , A. Alonso , A. Z. Beaton , M. S. Bittencourt , A. K. Boehme , A. E. Buxton , A. P. Carson , Y. Commodore‐Mensah , M. S. V. Elkind , K. R. Evenson , C. Eze‐Nliam , J. F. Ferguson , G. Generoso , J. E. Ho , R. Kalani , S. S. Khan , B. M. Kissela , K. L. Knutson , D. A. Levine , T. T. Lewis , J. Liu , M. S. Loop , J. Ma , M. E. Mussolino , S. D. Navaneethan , A. M. Perak , R. Poudel , M. Rezk‐Hanna , et al., Circulation 2022, 145, 153. 10.1161/CIR.0000000000001052 35078371

[2] M. R. Khazdair , M. Moshtagh , A. Anaeigoudari , S. Jafari , T. Kazemi , Food Sci. Nutr. 2024, 12, 3137. 38726397 10.1002/fsn3.4014 PMC11077248

[3] D. Zhao , J. Liu , M. Wang , X. Zhang , M. Zhou , Nat. Rev. Cardiol. 2019, 16, 203. 30467329 10.1038/s41569-018-0119-4

[4] J. E. Roeters van Lennep , L. S. Tokgözoglu , L. Badimon , S. M. Dumanski , M. Gulati , C. N. Hess , K. B. Holven , M. Kavousi , M. Kayikioglu , E. Lutgens , E. D. Michos , E. Prescott , J. K. Stock , A. Tybjaerg‐Hansen , M. J. H. Wermer , M. Benn , Eur. Heart J. 2023, 44, 4157. 37611089 10.1093/eurheartj/ehad472 PMC10576616

[5] M. Mehu , C. A. Narasimhulu , D. K. Singla , Antioxidants 2022, 11, 233. 35204116 10.3390/antiox11020233 PMC8868126

[6] L. Shen , W. Chen , J. Ding , G. Shu , M. Chen , Z. Zhao , S. Xia , J. Ji , FASEB J. 2023, 37, 22791. 10.1096/fj.202201486R 36723768

[7] G. Domschke , C. A. Gleissner , Cytokine 2019, 122, 154141. 28899579 10.1016/j.cyto.2017.08.021

[8] L. Liberale , F. Dallegri , F. Montecucco , F. Carbone , Thromb. Haemost. 2017, 117, 07. 10.1160/TH16-08-0593 27683760

[9] A V. Finn , M. Nakano , R. Polavarapu , V. Karmali , O. Saeed , X. Zhao , S. Yazdani , F. Otsuka , T. Davis , A. Habib , J. Narula , F D. Kolodgie , R. Virmani , J. Am. Coll. Cardiol. 2012, 59, 166. 22154776 10.1016/j.jacc.2011.10.852 PMC3253238

[10] J J. Boyle , M. Johns , J. Lo , A. Chiodini , N. Ambrose , P. C. Evans , J. C. Mason , D. O. Haskard , Arterioscler. Thromb. Vasc. Biol. 2011, 31, 2685. 21868703 10.1161/ATVBAHA.111.225813

[11] I. Tabas , K. E. Bornfeldt , Circ. Res. 2020, 126, 1209. 32324504 10.1161/CIRCRESAHA.119.315939 PMC7392397

[12] L. Shen , H. Li , W. Chen , Y. Su , J. Yu , M. Chen , G. Shu , E. Qiao , X. Guo , M. Xu , S. Xia , Z. Zhao , C. Lu , J. Ji , Biochim. Biophys. Acta Mol. Basis Dis. 2022, 1868, 166550. 36150660 10.1016/j.bbadis.2022.166550

[13] F. Wang , S. Zhang , R. Jeon , I. Vuckovic , X. Jiang , A. Lerman , C. D. Folmes , P. D. Dzeja , J. Herrmann , EBioMedicine 2018, 30, 303. 29463472 10.1016/j.ebiom.2018.02.009 PMC5953001

[14] S. Umar , K. Palasiewicz , M V. Volin , B. Romay , R. Rahat , C. Tetali , S. Arami , M. Guma , C. Ascoli , N. Sweiss , R K. Zomorrodi , L A. J. O'Neill , S. Shahrara , Cell. Mol. Life Sci. 2021, 78, 7693. 34705053 10.1007/s00018-021-03978-5 PMC8739866

[15] W. Zheng , M. Umitsu , I. Jagan , C. W. Tran , N. Ishiyama , M. BeGora , K. Araki , P S. Ohashi , M. Ikura , S. K. Muthuswamy , Nat. Cell Biol. 2016, 18, 1244. 27694890 10.1038/ncb3413

[16] G. J. Koelwyn , E. M. Corr , E. Erbay , K. J. Moore , Nat. Immunol. 2018, 19, 526. 29777212 10.1038/s41590-018-0113-3 PMC6314674

[17] D. Wang , V. Hiebl , D. Schachner , A. Ladurner , E. H. Heiss , A. G. Atanasov , V. M. Dirsch , Biochem. Pharmacol. 2020, 177, 114022. 32437644 10.1016/j.bcp.2020.114022

[18] M. A. Selak , S. M. Armour , E. D. MacKenzie , H. Boulahbel , D. G. Watson , K. D. Mansfield , Y. Pan , M. C. Simon , C. B. Thompson , E. Gottlieb , Cancer Cell 2005, 7, 77. 15652751 10.1016/j.ccr.2004.11.022

[19] S.‐J. Park , K.‐P. Lee , S. Kang , J. Lee , K. Sato , H. Y. Chung , F. Okajima , D.‐S. Im , Cell Signal 2014, 26, 2249. 25035231 10.1016/j.cellsig.2014.07.009

[20] Y. Bi , J. Chen , F. Hu , J. Liu , M. Li , L. Zhao , Neural Plast 2019, 21, 6724903. 10.1155/2019/6724903 PMC6409015 30923552

[21] J. Van den Bossche , J. Baardman , N. A. Otto , S. van der Velden , A. E Neele , S. M. van den Berg , R. Luque‐Martin , H. J. Chen , M. C. Boshuizen , M. Ahmed , M. A. Hoeksema , A. F. de Vos , M. P. de Winther , Cell Rep 2016, 17, 684. 27732846 10.1016/j.celrep.2016.09.008

[22] Q. Li , Q. Zhao , J. Zhang , L. Zhou , W. Zhang , B. Chua , Y. Chen , Li Xu , P. Li , Cell Rep. 2019, 28, 3406. 31553910 10.1016/j.celrep.2019.08.066

[23] B. Kahali , Y. Chen , M. F. Feitosa , L. F. Bielak , J. R. O'Connell , S. K. Musani , Y. Hegde , Y. Chen , L. C. Stetson , X. Guo , Y.‐P. Fu , A. V. Smith , K. A. Ryan , G. Eiriksdottir , A. T. Cohain , M. Allison , A. Bakshi , D. W. Bowden , M. J. Budoff , J. J. Carr , S. Carskadon , Y.‐D. I. Chen , A. Correa , B. F. Crudup , X. Du , T. B. Harris , J. Yang , S. L. R. Kardia , L. J. Launer , J. Liu , et al., J. Clin. Endocrinol. Metab. 2021, 106, 372. 33231259 10.1210/clinem/dgaa855 PMC7823249

[24] A. Tin , P. Balakrishnan , T. H. Beaty , E. Boerwinkle , R. C. Hoogeveen , J. H. Young , W. H. L. Kao , Diabet Med. 2016, 33, 968. 26433129 10.1111/dme.12971 PMC4819009

[25] S. Stender , E. Smagris , B. K. Lauridsen , K. F. Kofoed , B. G. Nordestgaard , A. Tybjærg‐Hansen , L. A. Pennacchio , D. E. Dickel , J. C. Cohen , H. H. Hobbs , Hepatology 2018, 67, 2182. 29266543 10.1002/hep.29751 PMC5991995

[26] X. Deng , C. Wang , Y. Xia , G. Yuan , Biomolecules 2022, 12, 1755. 36551183 10.3390/biom12121755 PMC9775135

[27] D. M. Waterworth , S. L. Ricketts , K. Song , L. Chen , J. H. Zhao , S. Ripatti , Y. S. Aulchenko , W. Zhang , X. Yuan , N. Lim , J. Luan , S. Ashford , E. Wheeler , E. H. Young , D. Hadley , J. R. Thompson , P. S. Braund , T. Johnson , M. Struchalin , I. Surakka , R. Luben , K.‐T. Khaw , S. A. Rodwell , R. J. F. Loos , S. M Boekholdt , M. Inouye , P. Deloukas , P. Elliott , D. Schlessinger , S. Sanna , et al., Arterioscler. Thromb Vasc. Biol. 2010, 30, 2264. 20864672 10.1161/ATVBAHA.109.201020 PMC3891568

[28] M. S. Semrau , G. Giachin , S. Covaceuszach , A. Cassetta , N. Demitri , P. Storici , G. Lolli , Nat. Commun 2022, 13, 6199. 36261419 10.1038/s41467-022-33693-z PMC9582199

[29] M. Inouye , S. Ripatti , J. Kettunen , L.‐P. Lyytikäinen , N. Oksala , P.‐P. Laurila , A. J. Kangas , P. Soininen , M. J. Savolainen , J. Viikari , M. Kähönen , M. Perola , V. Salomaa , O. Raitakari , T. Lehtimäki , M.‐R. Taskinen , M.‐R. Järvelin , M. Ala‐Korpela , A. Palotie , P I. W. de Bakker , PLoS Genet. 2012, 8, 1002907. 10.1371/journal.pgen.1002907 PMC3420921 22916037

[30] E. J. Benjamin , J. Dupuis , M. G. Larson , K. L. Lunetta , S. L. Booth , D. R. Govindaraju , S. Kathiresan , J. F. Keaney , M. J. Keyes , J.‐P. Lin , J. B. Meigs , S. J. Robins , J. Rong , R. Schnabel , J. A. Vita , T. J. Wang , P W. Wilson , P. A. Wolf , R. S. Vasan , BMC Med. Genet. 2007, 8, S11. 17903293 10.1186/1471-2350-8-S1-S11 PMC1995615

[31] Y. Zhang , W. Gan , C. Tian , H. Li , X. Lin , Y. Chen , J Diabetes 2013, 5, 275. 23343124 10.1111/1753-0407.12028

[32] S. Xue , Z. Su , D. Liu , Ageing Res. Rev. 2023, 90, 101993. 37379970 10.1016/j.arr.2023.101993

[33] A. K. Jha , S. C.‐C. Huang , A. Sergushichev , V. Lampropoulou , Y. Ivanova , E. Loginicheva , K. Chmielewski , K. M. Stewart , J. Ashall , B. Everts , E. J. Pearce , E. M. Driggers , M. N. Artyomov , Immunity 2015, 42, 419. 25786174 10.1016/j.immuni.2015.02.005

[34] Z. Tan , Na Xie , H. Cui , D. R. Moellering , E. Abraham , V. J. Thannickal , G. Liu , J. Immunol. 2015, 194, 6082. 25964487 10.4049/jimmunol.1402469 PMC4458459

[35] M. Nomura , J. Liu , I. I. Rovira , E. Gonzalez‐Hurtado , J. Lee , M. J. Wolfgang , T. Finkel , Nat. Immunol. 2016, 17, 216. 26882249 10.1038/ni.3366 PMC6033271

[36] L. Hertz , L. Peng , G. A. Dienel , J. Cereb. Blood Flow Metab. 2007, 27, 219. 16835632 10.1038/sj.jcbfm.9600343

[37] A. Rossi , C. Simeoli , R. Pivonello , M. Salerno , C. Rosano , B. Brunetti , P. Strisciuglio , A. Colao , G. Parenti , D. Melis , T. G. J. Derks , Rev. Endocr. Metab. Disord. 2024, 25, 707. 38556561 10.1007/s11154-024-09880-2 PMC11294274

[38] Q. Chen , J. Lv , W. Yang , B. Xu , Z. Wang , Z. Yu , J. Wu , Y. Yang , Y. Han , Theranostics 2019, 9, 6424. 31588227 10.7150/thno.35528 PMC6771242

[39] K. S. Chun , J. H. Jang , D. H. Kim , Cells 2020, 9, 2202.33003453

[40] R. Li , X. Li , J. Zhao , F. Meng , C. Yao , E. Bao , N. Sun , X. Chen , W. Cheng , H. Hua , X. Li , B. Wang , H. Wang , X. Pan , H. You , J. Yang , T. Ikezoe , Theranostics 2022, 12, 976. 34976224 10.7150/thno.63751 PMC8692896

[41] T. Xia , M. Zhang , W. Lei , et al., Front. Immunol. 2023, 14, 1160719. 37081874 10.3389/fimmu.2023.1160719 PMC10110879

[42] H. Gotoh , T. A. Chimhanda , T. Nomura , K. Ono , FEBS Lett. 2022, 596, 2940. 36050761 10.1002/1873-3468.14489

