# Supplementary material for: PPP1R3B Suppresses Atherosclerosis by Promoting the M2 Polarization of Macrophages Through Glycogen Metabolic Reprogramming
Source: Adv Sci (Weinh). 2025 Sep 23;12(41):e06345. doi: 10.1002/advs.202506345 (PMC12591178; doi:10.1002/advs.202506345)
Supplement: Supplementary file 1 — Supporting Information [file ADVS-12-e06345-s004.docx]

**Supplementary Materials**

***PPP1R3B* suppresses atherosclerosis by promoting the M2 polarization of macrophages through glycogen metabolic reprogramming**

*Lin Shen*^±^*, Junchao Yu*^±^*, Weiqian Chen*^±^*, Yanran Bi, Zhangyu Yang, Chenying Lu, Chengli Jiang, Yang Yang, Minjiang Chen, Jianhua Zou^*^, Lingchun Lv^*^, Xiaoyuan Chen^*^, Jiansong Ji^*^*

L. Shen, J. Yu, W. Chen, Y. Bi, Z. Yang, C. Lu, C. Jiang, Y. Yang, M. Chen, J. Ji

Zhejiang Key Laboratory of Imaging and Interventional Medicine, Zhejiang Engineering Research Center of Interventional Medicine Engineering and Biotechnology, Key Laboratory of Precision Medicine of Lishui City, The Fifth Affiliated Hospital of Wenzhou Medical University, Lishui 323000, China. Email: [*lvlingchun@medmail.com.cn*](mailto:lvlingchun@medmail.com.cn)*,* [*jjstcty@wmu.edu.cn*](mailto:jjstcty@wmu.edu.cn).

J. Yu, C. Lu, C. Jiang, Y. Yang, M. Chen, J. Ji

Department of Radiology, Lishui Hospital of Zhejiang University, School of Medicine, Lishui, 323000, China

L. Lv,

Department of Cardiology, the Fifth Affiliated Hospital of Wenzhou Medical University, Lishui 323000, China.

L. Shen, W. Chen, Y. Bi, C. Lu, Y. Yang, M. Chen, L. Lv, J. Ji

Clinical College of The Affiliated Central Hospital, School of Medicine, Lishui University, Lishui, 323000, China

J. Zou. X. Chen

Departments of Diagnostic Radiology, Surgery, Chemical and Biomolecular Engineering, and Biomedical Engineering, Yong Loo Lin School of Medicine and College of Design and Engineering, National University of Singapore, Singapore, 119074, Singapore. Email: [*zoujh-93@nus.edu.sg*](mailto:zoujh-93@nus.edu.sg)*,* [*chen.shawn@nus.edu.sg*](mailto:chen.shawn@nus.edu.sg)*.*

Nanomedicine Translational Research Program, Yong Loo Lin School of Medicine, National University of Singapore, Singapore 117597, Singapore

Theranostics Center of Excellence (TCE), Yong Loo Lin School of Medicine, National University of Singapore, 11 Biopolis Way, Helios, Singapore 138667

X. Chen

Clinical Imaging Research Centre, Centre for Translational Medicine, Yong Loo Lin School of Medicine, National University of Singapore, Singapore 117599, Singapore

Department of Pharmacy and Pharmaceutical Sciences, National University of Singapore, Lower Kent Ridge Road, 4 Science Drive 2, 117544, Singapore.

^±^These authors contributed equally to this work.

**Materials and Methods**

**Collection of human atherosclerotic plaque samples**

The study protocol for collecting human atherosclerotic plaque samples was approved by the Research Ethics Committee of the Fifth Affiliated Hospital of Wenzhou Medical University (22024-119) and complied with the Declaration of Helsinki. Atherosclerotic plaques were collected from 20 patients diagnosed with carotid AS who underwent carotid endarterectomy at the Fifth Affiliated Hospital of Wenzhou Medical University. Each patient had previously been diagnosed with a transient ischemic attack or cerebral infarction and exhibited symptoms such as vertigo or cognitive impairment. Informed consent was obtained from all participants. They were offered no compensation for their involvement in this study.

**Cell culture and treatment**

Bone marrow-derived MΦs (BMDMs) were collected from 6–8-week-old male C57BL/6J mice obtained from the SLAC laboratory Animal Co., Ltd. (Shanghai, China). The BMDMs were incubated with 20 ng mL^−1^ of MΦ colony-stimulating factor 1 (CSF1/M-CSF) for three days to induce differentiation.

THP-1-derived MΦs were created by culturing human leukemia monocytic THP-1 cells in six-well tissue culture plates at a density of 5 × 10^4^ cells per well using RPMI 1640 medium supplemented with 10% fetal bovine serum. The THP-1 cells were incubated with phorbol 12-myristate 13-acetate (PMA; 100 ng mL^−1^) for 72 hours to induce their differentiation into MΦs.

After obtaining the above-differentiated MΦs, stimulation with LPS (100 ng mL^−1^) and IL-4 (20 ng mL^−1^) can induce their polarization towards the M1 or M2 phenotype, respectively. The murine RAW264.7 cell line, as a commonly used macrophage cell line, can be stimulated using the same method. Specific details on the duration of differentiation and the concentration of the reagent used in the assay are provided in supplementary Table 2.

**Transfection vector**

Transfection vectors that knockdown (sh*PPP1R3B*; 30710GX-LV) or overexpress (ov*PPP1R3B*; 30708GV-LV) *PPP1R3B* and adeno-associated virus (AAV)-*PPP1R3B* (GM-AA-129307) were created by Genomeditech (Shanghai, China). Various viral transfection methods for cells and mice were performed according to the protocols provided by Genomeditech.

**Ethics approval and consent to participate**

All animal procedures were approved by the Experimental Animal Ethics Committee of Wenzhou Medical University (Protocol number. xmsq2024-0003). The animals were housed in standard environment and were managed in accordance with the requirements of the "Regulations on the Administration of Experimental Animals." After the experiment, the animals were euthanized by overdose anesthesia (Isoflurane, 5%, 5-10min). The entire experiment adhered to the "3R" principlesto ensure animal welfare.

**Animal model**

Apolipoprotein E (*Apoe*)^−/−^ mice (C57BL/6J background) were obtained from the SLAC laboratory Animal Co., Ltd. (Shanghai, China). To establish the *in vivo* atherosclerotic mouse model, 4–6-week-old male *Apoe*^−/−^ mice were fed either a high-fat diet (HFD) containing 1.25% cholesterol and 40% (w/w) fat or a normal diet containing 10% (w/w) fat for three months. Mice that were intolerant to the HFD were excluded from this study. The adeno-associated virus (AAV)-protein phosphatase 1 regulatory subunit 3B (*PPP1R3B*)-transfected mice were constructed by administering 5 × 10^11^ vector genome copies of AAV-*PPP1R3B* (ov*PPP1R3B*) or an AAV-Ctrl (Ctrl) to 4–6-week-old male *Apoe*^−/−^ mice. After transfection, the mice were maintained on an HFD for three months to assess the effects of the genetic modification.

To construct the ov*PPP1R3B*+STAT3 agonist 1 group of mice, the STAT3 agonist 1 (HY-P1061A, MedChemExpress) was dissolved in 5% dimethyl sulfoxide (DMSO) and administered intraperitoneally (i.p.) at a dosage of 1 mg kg^−1^ to *Apoe*^−/−^ mice transfected with ov*PPP1R3B.* To construct the ov*PPP1R3B*+rotenone group of mice, rotenone (HY-B1756, MedChemExpress) was dissolved in DMSO and administered i.p. at a dosage of 2 mg kg^−1^ to *Apoe*^−/−^ mice transfected with ov*PPP1R3B*. To construct the ov*PPP1R3B*+STAT3 agonist 1+rotenone mouse group, *Apoe*^−/−^ mice transfected with ov*PPP1R3B* were administrated with STAT3 agonist 1, and rotenone was dissolved in DMSO before they were co-administered i.p. at dosages of 1 (in 5% ethanol) and 20 mg kg^−1^(in 5% DMSO), respectively. All mice were euthanized through terminal inhalation of a 5% isoflurane/oxygen mixture.

The investigators were blinded to the treatment groups during outcome assessments following these procedures.

**Relative cell treatment**

BMDMs and THP-1-derived MΦs with *PPP1R3B* knockdown or overexpression were created by transfecting them with lentivirus carrying either *PPP1R3B*-small hairpin RNA (sh*PPP1R3B*) or *PPP1R3B*-ovRNA (ov*PPP1R3B*) for 24 hours, respectively. After transfection, *PPP1R3B* mRNA and protein levels in the MΦs were quantitatively analyzed using real-time PCR and western blotting, respectively. Then, both THP-1 cells and BMDMs transfected with sh*PPP1R3B* or ov*PPP1R3B* were differentiated into M1 and M2 MΦs using lipopolysaccharide (LPS; 100 ng mL^−1^) or interleukin 4 (IL4; 20 ng mL^−1^), respectively.

**High-throughput sequencing and bioinformatics analysis of MΦs in human atherosclerotic plaques**

To investigate the immunometabolic signaling pathways that regulate MΦ polarization in human atherosclerotic plaques, differentially expressed genes (DEGs) associated with MΦ polarization in these plaques were identified using the Gene Expression Omnibus database. Then, the signaling pathways influencing MΦ polarization, driven by these DEGs in human atherosclerotic plaques, were explored through gene set enrichment analysis (GSEA), weighted gene co-expression network analysis (WGCNA), gene ontology (GO) enrichment analysis, Kyoto Encyclopedia of Genes and Genomes (KEGG) enrichment analysis, and Metascape analysis.

Next-generation RNA sequencing (RNA-seq) was used to explore the key targets of *PPP1R3B* in regulating MΦ polarization. First, total RNA was extracted from M1 and M2 MΦs transfected with sh*PPP1R3B* or ov*PPP1R3B* using the RNasy Mini Kit with DNase treatment (Qiagen). Chain-specific libraries were constructed with the KAPA mRNA HyperPrep Kit (KAPA BioSystems). Next, the RNA samples were sequenced on a HiSeq 4000 instrument (Illumina). Then, the reads were aligned to build mm10 of the mouse genome using STAR (version 2.5.2b), and the BAM files were indexed and filtered with SAMtools. Raw tag counts and reads per kilobase per million mapped reads values were aggregated using the analyzeRepeats.pl script from HOMER2. DEG analysis was performed using the DESeq2 package in the R statistical software (version 4.1.2; R Core Team, 2021). Reactome pathway enrichment analysis of significant DEGs (adjusted *p*-value ≤0.05) was performed using the ReactomePA package in R. Redundant pathways representing parent functionality were extracted from the significantly enriched pathways list. Ridge plots visualizing the summarized pathway list were created by extracting gene IDs and plotting fold change distributions using custom scripts. Heatmaps were created with the ggplot2, ggrepel, and pheatmap packages in R.

GSEA was performed using the GSEA software (version 3.0; http://www.broad.mit.edu/gsea/) and the Molecular Signature Database (version 6.2) using the GSEA pre-ranked function. The DEG analysis outputs from DESeq2 were first used to generate the ranked list file. Then, the data were integrated and analyzed using the multiGSEA package in R.

WGCNA was used to identify DEGs involved in *PPP1R3B* regulation of MΦ polarization. The R package WGCNA was used to analyze the co-expression modules of DEGs. The WGCNA parameters of the soft threshold power of the adjacency matrix and the criteria of the coefficient of determination were defined according to the approximate scale-free topology preconditions and the criteria of cut-off of ≥30 genes and cut height of 0.15. The adjacency matrix dissimilarity was 0.2. Then, the WGCNA modules (co-expression network) of eigengenes were identified, and the networks correlated with the polarization of M1/M2 MΦs were identified based on a stability correlation *p* ≤ 0.05. The modules with genes significantly correlated with M1/M2 MΦ polarization (Pearson’s correlation coefficient [*r*] ≥ 0.6; Cd treatment, Cd accumulation, and different tissue nodes) were retained for further analyses.

Pathway enrichment analysis and gene network reconstruction were performed using the Metascape tool (http://metascape.org) with default parameters. The pathway and enrichment analyses were conducted using the gene set obtained from the WGCNA analysis, selecting the genomics sources KEGG Pathway, GO Biological Processes, Reactome Gene Sets, Canonical Pathways, and CORUM.

**Cell function evaluation**

Mitochondrial function was assessed using a mitochondrial membrane potential assay kit with the JC-1 dye (Beyotime, Shanghai, China). The cells were seeded in six-well plates at a density of 5 × 10^4^ cells per well. Next, 1 mL of cell culture medium and 1 mL of the JC-1 staining solution were added. Then, the cells were incubated in a cell culture incubator at 37°C with 5% CO_2_ and saturated humidity for 20 minutes. After incubation, the supernatant was removed, and the cells were washed twice with JC-1 staining buffer. Finally, 2 mL of cell culture medium was added to the cells, which were then examined under a laser confocal microscope (Leica).

A 5-ethynyl-2’-deoxyuridine (EdU) cell proliferation detection kit (Beyotime, Shanghai, China) was used to assess the effect of *PPP1R3B* on MΦs proliferation. Approximately 1 × 10⁵ bone marrow–derived macrophages (BMDMs) were seeded in 6-well plates and transfected with either sh*PPP1R3B* or ov*PPP1R3B*. Cells were subsequently polarized into M2 phenotypes using IL-4 (20 ng mL^−1^), followed by incubation with 1 mL of EdU solution. After two hours, cells were fixed with 4% paraformaldehyde (PFA), stained with Apollo dye to detect EdU incorporation, and counterstained with Hoechst-33342 to visualize nuclei. Fluorescence images were captured using a Nikon microscope, and the proportion of EdU^+^ cells was quantified. All

experiments were performed in biological triplicates (*n* = 3), and quantitative data are presented as mean ± standard deviation (SD). Statistical significance was evaluated using one-way ANOVA followed by Tukey’s post hoc test; *p*-values < 0.05 were considered statistically significant.

A cell cycle and apoptosis analysis kit (Beyotime, Shanghai, China) was used to explore the regulatory effect of *PPP1R3B* on the MΦ cell cycle. M1 and M2 MΦs transfected with sh*PPP1R3B* or ov*PPP1R3B* were harvested and fixed overnight in 70% ethanol at −20°C. Next, the fixed cells were washed with phosphate-buffered saline (PBS) to remove the ethanol and incubated with a staining buffer containing RNase A at 37°C for 30 minutes to digest RNA. Then, the cell pellet was slowly and thoroughly resuspended in a propidium iodide staining solution for DNA staining. Red fluorescence was detected using a flow cytometer at an excitation wavelength of 488 nm. Finally, the flow cytometer data were analyzed using FlowJo software (version 10; Treestar) to determine the proportion of cells in each cell cycle phase.

A cell clonal formation assay was used to assess the impact of *PPP1R3B* on MΦ proliferation. First, M2 MΦs transfected with sh*PPP1R3B* or ov*PPP1R3B* were plated at a density of 700 cells per well in a six-well plate to promote single-cell growth and cultured at 37°C in a 5% CO_2_ incubator for two weeks, with periodic replacements of the culture medium. After cultivation, the cells were fixed with 4% PFA for 15 minutes and stained with crystal violet. Finally, clusters containing more than 50 cells were counted under a microscope to quantify colony formation.

**Flow cytometry analysis**

The M1/M2 polarization of BMDMs cultured *in vitro* or within aortic single-cell suspensions from treated mice was assessed using flow cytometry. Aortic single-cell suspensions were prepared by euthanizing mice from each group and isolating and extracting their aortas. Then, the aortas were digested in a PBS solution containing 1 mg mL^−1^ collagenase A, 1 mg mL^−1^ collagenase B, 60 U mL^−1^ hyaluronidase, and 0.1 mg mL^−1^ DNase I at 37°C for one hour. After digestion, the suspension was filtered through a 70 µm cell strainer.

Next, BMDMs and aortic cell suspensions were analyzed by flow cytometry. The cells were first blocked with FcR Blocking Reagent (1 μg test^−1^; BioLegend) and then immunostained with the following antibodies: AF488-labeled anti-mouse adhesion G protein-coupled receptor E1 (ADGRE1 or F4/80; 1 μg test^−1^; 123119, BioLegend), PerCP-Cy5.5 conjugated anti-mouse integrin alpha M (ITGAM/CD11b; 0.25 μg test^−1^; 101227, BioLegend), and PE-labeled anti-mouse CD86 antigen (CD86; 1 μg test^−1^; 105007, BioLegend). After staining, the cells were incubated with 500 μL of fixation buffer (BioLegend) and 500 μL of Intracellular Staining Permeabilization Wash Buffer (BioLegend). Then, the cells were immunostained with an APC-labeled anti-mouse antibody against mannose receptor C-type 1 (MRC1/CD206; 0.5 μg test^-1^; 141707, BioLegend), following the manufacturer’s instructions. Finally, flow cytometry was performed, and the data were analyzed using FlowJo software.

**Binding site prediction and dual-luciferase reporter gene detection**

Potential STAT3 binding sites in the mouse *PPP1R3B* or peroxisome proliferator-activated receptor gamma (PPARγ) genes were predicted using the position weight matrix algorithm from JASPAR. This analysis involved scanning their promoter regions, which were defined as extending from −2000 to +500 bp relative to their transcriptional start site.

A dual-luciferase reporter gene detection plasmid and cell line were prepared using the experimental plasmid pGL4.10 (H352; OBiO Tech), pGL4.10-*PPP1R3B* promoter (wildtype; H30452; OBiO Tech), pGL4.10-*PPP1R3B* promoter (mutant; H30453; OBiO Tech), pCDNA3.1^+^-3×FLAG-P2A-EGFP (H2713; OBiO Tech), pCDNA3.1^+^-STAT3-3×FLAG-P2A-EGFP (H34381; OBiO Tech), pGL4.10-*PPARG* promotor (wildtype; H30450; OBiO Tech), pGL4.10-*PPARG* promotor (mutant; H30451; OBiO Tech), pcDNA3.1^+^-3×FLAG-P2A-EGFP (H2713; OBiO Tech), pcDNA3.1^+^-STAT3-3×FLAG-P2A-EGFP (H24381; OBiO Tech), and pRL-CMV (H321; Promega). The 293T cell line was obtained from the Cell Bank of the Chinese Academy of Sciences and cultured at 37°C with 5% CO_2_. Then, dual-luciferase reporter gene detection was performed.

**Integrating immunoprecipitation (IP) with mass spectrometry (MS) for protein interaction analysis**

MΦs transfected with ov*PPP1R3B* were harvested and lysed using a buffer containing protease inhibitors to extract proteins. Pre-clearing with appropriate protein A/G beads removed non-specifically bound proteins from the lysate. Next, a specific anti-*PPP1R3B* antibody (ab235049; Abcam) was added to the lysate and incubated overnight to allow binding to the target protein. Then, protein A/G beads were added to capture the antibody-protein complex, followed by incubation to form the immunocomplex. Next, the beads were washed multiple times with lysis buffer to eliminate non-specifically bound proteins. Then, the immunocomplex was eluted from the beads using a sodium dodecyl sulfate (SDS) sample buffer, and the eluted proteins were heated or boiled to dissociate the complexes. Finally, the dissociated protein samples were analyzed by MS to identify co-precipitated protein components.

**Western blotting and co-IP analysis**

Cells or tissues were harvested and homogenized with RIPA buffer containing protease and phosphatase inhibitors. Next, mitochondrial proteins were extracted and prepared using a Mitochondrial Extraction Kit (Solarbio, Shanghai, China), and nuclear proteins were extracted and prepared using a Nuclear Protein Extraction Kit (Solarbio, Shanghai, China). Then, the proteins were separated via SDS-polyacrylamide gel electrophoresis and transferred to polyvinylidene difluoride membranes (Millipore, Billerica, MA, USA). After blocking with 5% skim milk at room temperature (RT) for one hour and washing three times with tris-buffered saline containing 1% Tween 20, the membranes were incubated with the primary antibody (1:1000) in an antibody dilution buffer at 4°C on a shaker overnight, followed by the secondary antibody (1:10000) in an antibody dilution buffer on a shaker at RT for one hour. Protein bands were detected using an Invitrogen iBright CL750 imager (Thermo Fisher Scientific). Densitometry of the western blotting was analyzed and normalized to actin beta (ACTB) using the ImageJ software (version 1.8.0).

The following primary and secondary antibodies were used: anti-*PPP1R3B* (ab235049; Abcam), anti-arginase 1 (ARG1; 16001-1-AP; Proteintech), anti-nitric oxide synthase 2 (NOS2/iNOS; ab178945; Abcam), anti-CD206 (18704-1-AP; Proteintech), anti-catenin beta 1 (CTNNB1; ab32572; Abcam), anti-glycogen synthase kinase 3 beta (GSK3B/GSK-3β; ab32391; Abcam), anti-phosphorylated (p)-GSK-3β (9336s; Cell Signaling Technology), anti-glycogen synthase 2 (GYS2; 22371-1-AP; Proteintech), anti-p-GYS2 (3891s; Cell Signaling Technology), anti-glycogen phosphorylase L (PYGL; ab198268; Abcam), anti-p-PYGL (ab227043; Abcam), anti-PPARG coactivator 1 alpha (PPARGC1A/PGC-1α; ab12348; Abcam), and ACTB (sc-8432; Santa Cruz Biotechnology) as primary antibodies, and horseradish peroxidase (HRP)-conjugated anti-mouse IgG (7076S; Cell Signaling Technology) and HRP-conjugated anti-rabbit IgG (7074S; Cell Signaling Technology) as secondary antibodies. Band densities were quantified using the ImageJ software (version 1.8.0).

Following treatment, cell extracts were prepared and incubated with the indicated antibodies at 4°C overnight for co-IP assays. Next, the proteins were immunoprecipitated with Protein A+G Agarose at 4°C for two hours. Then, the IP samples were immunoblotted for co-precipitated protein detection. Total lysates were subjected to western blotting as input controls. Protein interactions were quantified using the ImageJ software (version 1.8.0).

**GST pull-down assay**

Initially, purified GST-STAT3 was obtained. Subsequently, to achieve the phosphorylated form of GST-STAT3, the essential components for phosphorylation, namely JAK2 kinase (20ng μL^−1^), ATP (50 μM ), and Mg2^+^ (5 mM), were introduced.To measure the binding affinity between *PPP1R3B* and STAT3(p) in vitro, GST-*PPP1R3B*, His-STAT3(p) and GST were added to the reaction mix and incubated further at 4 ℃ for 1 h with gentle rotation. The supernatant was then incubated with glutathione beads bound to GST-*PPP1R3B* or GST alone as a control (4 ℃, 18 h). After centrifugation (800 ×g, 1 min, 4 ℃), the beads were washed twice with PBS at 4 ℃. The pull-down proteins were eluted by boiling the beads in sample loading buffer for 5 min, followed by SDS-PAGE and Western blot analysis to detect protein interactions.

**Periodic acid-Schiff (PAS) staining**

The glycogen content of cells or 5-µm thick aortic sections was determined using a PAS Staining Kit (Beyotime, Shanghai, China). Glycogen was oxidized using a periodic acid solution and then stained with Schiff’s reagent at 37°C in the dark for one hour. Then, the stained cells or 5-μm thick aortic sections were observed under an inverted fluorescence microscope.

**Oil Red O (ORO) staining**

The cells were seeded at a density of 5 × 10^5^ cells per well in six-well plates. Once adhered, they were incubated with the polarization inducers LPS (100 ng mL^−1^; for M1 polarization) or IL4 (20 ng mL^−1^; for M2 polarization) for 24 hours. Next, the cells were fixed with 4% PFA for 15 minutes, stained with ORO solution for 10–20 minutes, and then briefly washed with PBS for 20 seconds. Then, adequate PBS was added to cover the cells completely. Finally, the stained cells were observed and imaged using an inverted fluorescence microscope.

After each treatment, mice were sacrificed and fixed in the supine position. Their thoracic cavity was opened immediately and perfused with saline through the left ventricle for two minutes. The whole aorta from the heart to the branch of the inferior iliac artery was quickly separated and stored in 4% PFA before detection. The atherosclerotic lesions were observed by removing the aorta from the 4% PFA fixation solution, rinsing them three times with PBS, and then opening them longitudinally or preparing 5-μm thick slices. ORO staining was performed to quantify atherosclerotic lesions in the aortas. The percentage of the area that was ORO^+^ was determined using the ImageJ software (version 1.8.0).

**Hematoxylin and eosin (H&E) staining**

All mice were euthanized at 16 weeks of age under sodium pentobarbital anesthesia. The entire aorta was excised, and the surrounding adipose and connective tissues were carefully removed. Next, the aortas were fixed overnight in 4% PFA at 4°C. Then, the formalin-fixed tissues were paraffin-embedded at the Histopathology Core Facility. Finally, 5-µm thick sections were prepared and stained with H&E for histological examination.

**Immunofluorescence staining**

Aortic root cryosections were blocked using 1% bovine serum albumin containing 0.1% Triton X-100 for one hour at RT. Next, the sections were incubated overnight at 4°C with a cocktail of primary antibodies (1:100): anti-*PPP1R3B* (ab235049; Abcam), anti-platelet/endothelial cell adhesion molecule 1 (PECAM1/CD31; ab9498; Abcam), anti-actin alpha 2, smooth muscle, aorta (ACTA2/α-SMA; A2547, MilliporeSigma), anti-F4/80 (ab6640; Abcam), anti-CD86 molecule (CD86; ab239075; Abcam), and anti-CD206 (ab64693; Abcam). Following primary antibody incubation, the sections were washed and incubated with a secondary antibody cocktail (ab6721 and ab6728; Abcam) for two hours at 4°C. Then, an anti-fade mounting medium containing 4’,6-diamidino-2-phenylindole (DAPI) was applied to the sections for five minutes. Finally, the fluorescently labeled slides were scanned using a laser scanning confocal microscope (Leica).

**Functional transmission electron microscopy (TEM)**

Intracellular glycogen accumulation in MΦs was visualized by fixing cells transfected with sh*PPP1R3B* or ov*PPP1R3B* and aortic plaque tissue from *Apoe*^−/−^ mice transduced with AAV-*PPP1R3B* with 2.5% glutaraldehyde at RT for one hour, followed by a secondary fixation with 1% osmium tetroxide at 4°C for one hour to enhance electron density. Next, the samples were progressively dehydrated using a graded series of ethanol concentrations (typically from 50% to 100%). After dehydration, the samples were embedded in a resin (typically Epon or Araldite). Then, ultra-thin sections approximately 70 nm thick were prepared using an ultramicrotome. The sections were stained with heavy metal salts, such as lead uranate and lead citrate, to improve contrast. These prepared samples were then examined under a TEM to observe the ultrastructural details of the cells.

**Pimonidazole adduct straining**

Using the Hypoxyprobe Kit (Mkbio, Shanghai, China), in vitro, cells were co-incubated with pimonidazole HCl (100 μM) for 1 hour. After fixation with 4% PFA, the cells were incubated with a fluorescent hypoxia probe antibody for 1 hour and kept on standby. In vivo, pimonidazole HCl was first administered via tail vein injection at a dose of 60 mg kg^−1^. The mice were allowed to move freely for 1 to 2 hour. After euthanasia, the aorta tissues were extracted and subjected to cryosectioning. Once the sections were prepared, they were first blocked with 5% BSA for 30 minutes, followed by incubation with the fluorescently labeled probe antibody overnight at 4 °C. Finally, the sections were imaged using a confocal microscope.

**Triple-Oxygen-State Immunofluorescence Assay for pSTAT3/PPP1R3B Localization in Polarized Macrophages**

M2-polarized THP-1 macrophages (induced by PMA + IL-4) were exposed to normoxia (20% O₂), hypoxia (1% O₂, 12 h), or reoxygenation (6 h normoxia post-hypoxia). Cells were fixed, permeabilized, and co-stained with anti-pSTAT3 (1:200) and anti-PPP1R3B (1:100) antibodies, followed by Alexa Fluor-conjugated secondaries and DAPI. Confocal microscopy images were quantitatively analyzed using ImageJ.

**Statistical analysis**

Statistical significance was defined as **p* < 0.05, with thresholds denoted as follows: **p* < 0.05, ***p* < 0.01, ****p* < 0.001, and *****p* < 0.0001. Data were analyzed using GraphPad Prism version 10.0 (GraphPad Software, San Diego, CA, USA). Prior to analysis, datasets were filtered to exclude outliers and standardized to ensure normality and homogeneity of variance. Intergroup differences were assessed using unpaired two-tailed Student’s t-test for comparisons between two groups or one-way analysis of variance (ANOVA) followed by Tukey’s post hoc test for multigroup comparisons. All experiments included a minimum of three independent biological replicates. Results are presented as mean ± standard deviation (SD), and significance levels are explicitly indicated in figures.

**
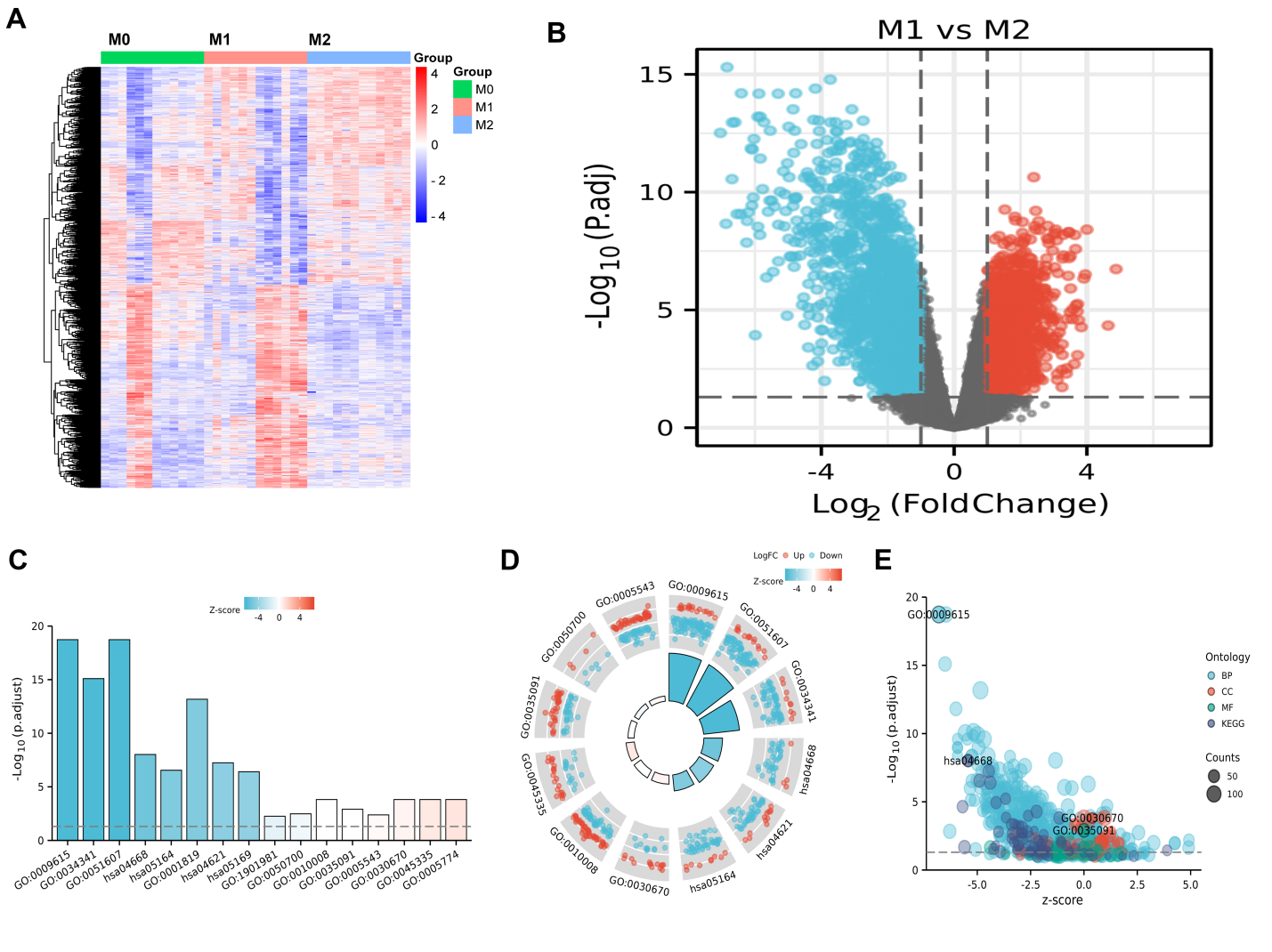
**

**Supplementary Fig. 1**. **(A)** heatmap and **(B)** volcano plot of DEGs upregulated (red) or downregulated (blue) in human AS plaques in the MΦ-related dataset GSE57614. **(C)** KEGG functional enrichment, **(D)** GO chord plot, **(E)** bubble plot in human AS-related MΦs.

**
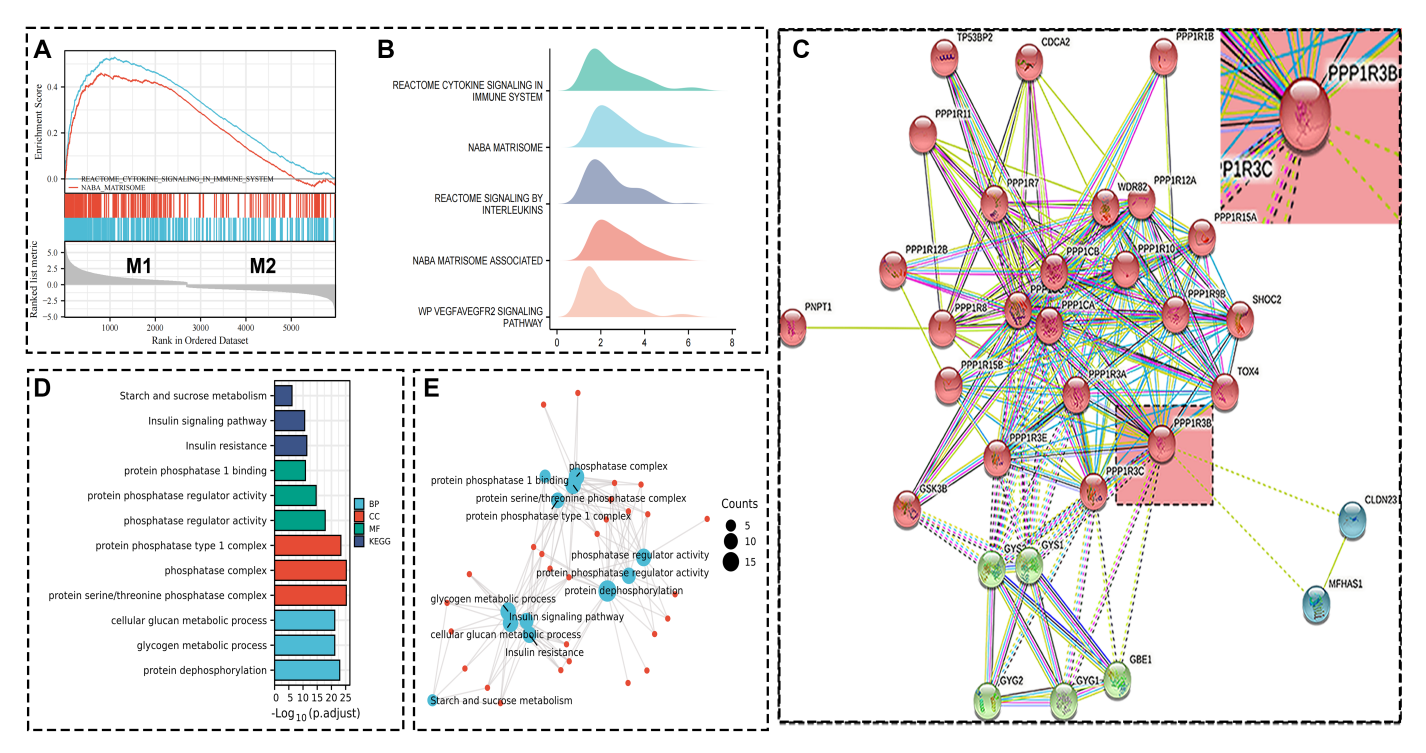
Supplementary Fig. 2**. **(A)** GSEA, and **(B)** peak map of DEGs in human AS-related MΦs. **(C)** Protein-protein interaction analysis of *PPP1R3B*. **(D)** KEGG functional enrichment analyses of *PPP1R3B*. (F) Cytoscape analyses of *PPP1R3B*.


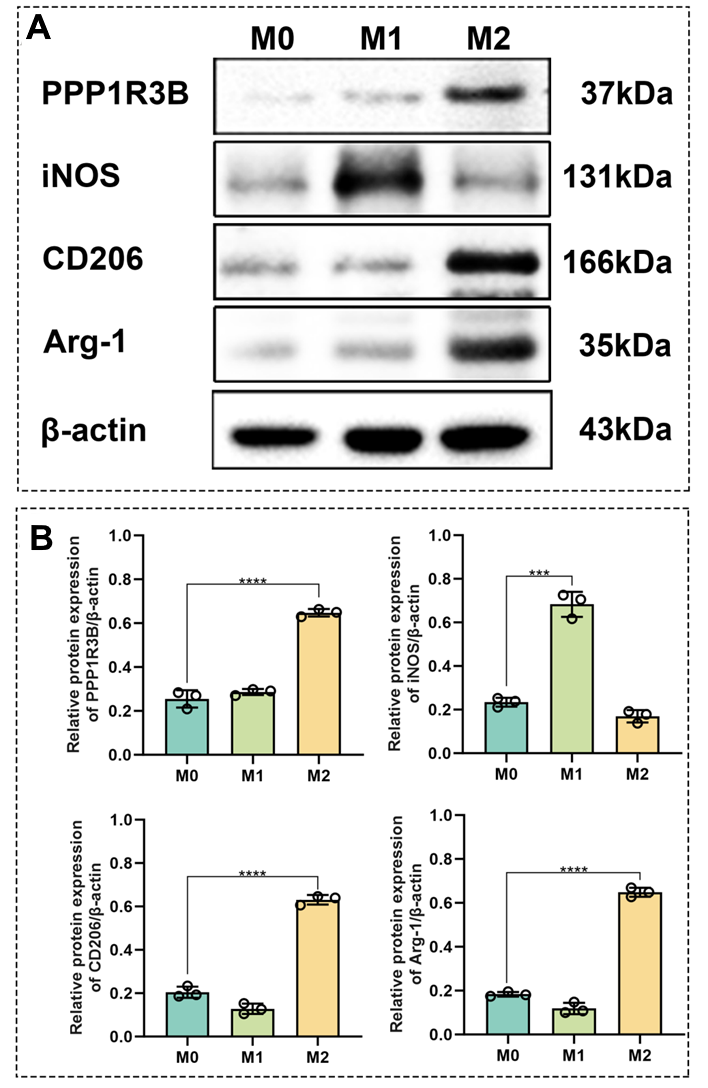


**Supplementary Fig. 3**. **(A)** Western blots of ***PPP1R3B***, iNOS, CD206, and ARG1 in different MΦ phenotypes (M0, M1, and M2); actin beta (ACTB) was used as the loading control (*n* = 3 biological replicates). **(B)** Statistical histogram of (A).The data are presented as the mean ± SEM across three biologically independent samples. Significance: **p* < 0.05; ***p* < 0.01; ****p* < 0.001; *****p* < 0.0001.


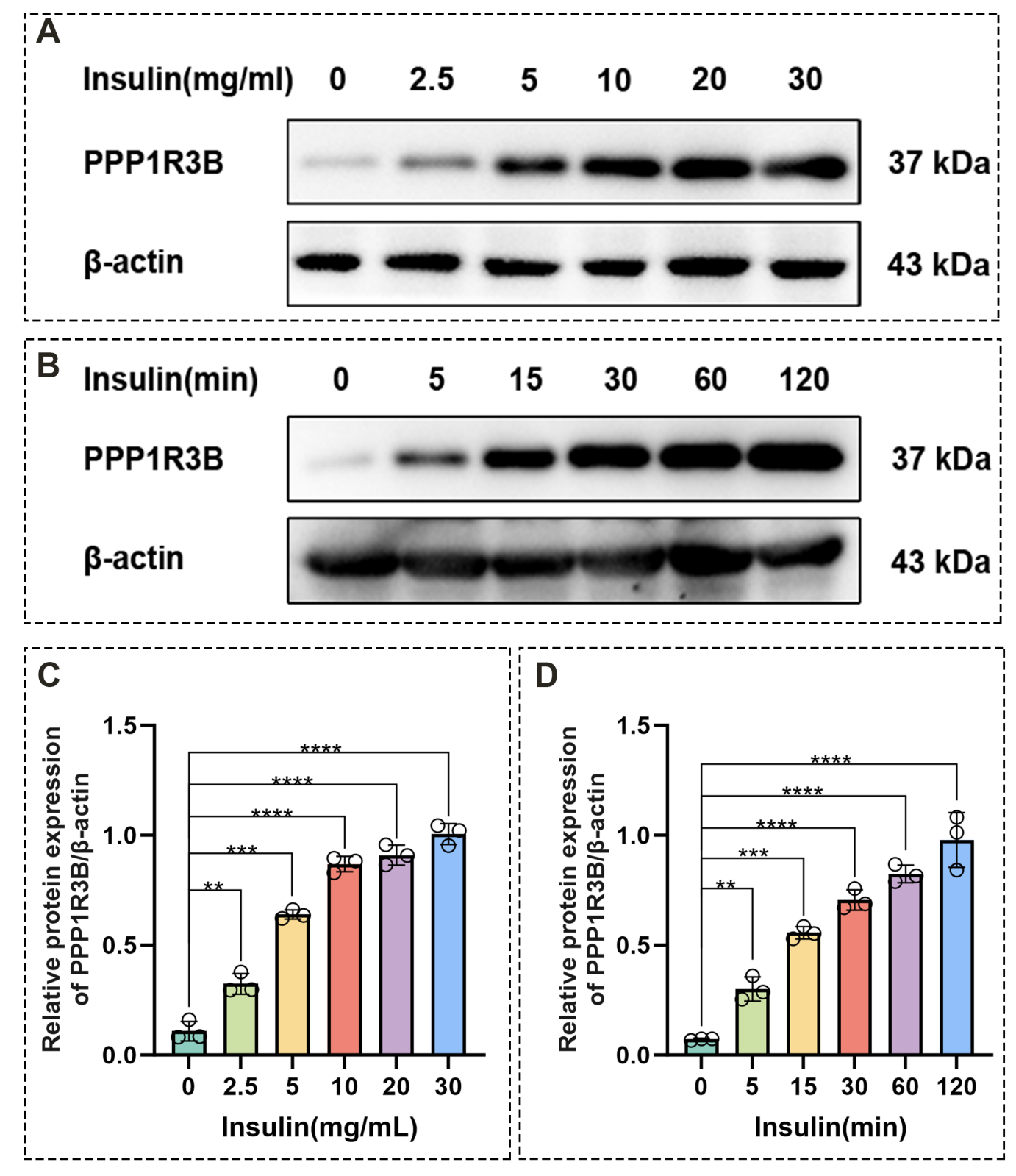
**Supplementary Fig. 4**. **(A)** Western blots of *PPP1R3B* expression in THP-1 cells after incubation with different insulin concentrations (0, 2.5, 5, 10, 20, and 30 mg mL^−1^) for 120 minutes. **(B)** Western blot of *PPP1R3B* expression in THP-1 cells induced with 30 mg mL^−1^ insulin at different time (0, 5, 15, 30, 60, and 120 minutes); ACTB was used as the loading control (*n* = 3 biological replicates). **(C)** Statistical histogram of (A). **(D)** Statistical histogram of (B). Data are presented as the mean ± SEM across three biologically independent samples. Significance: **p* < 0.05; ***p* < 0.01; ****p* < 0.001; *****p*<0.0001.


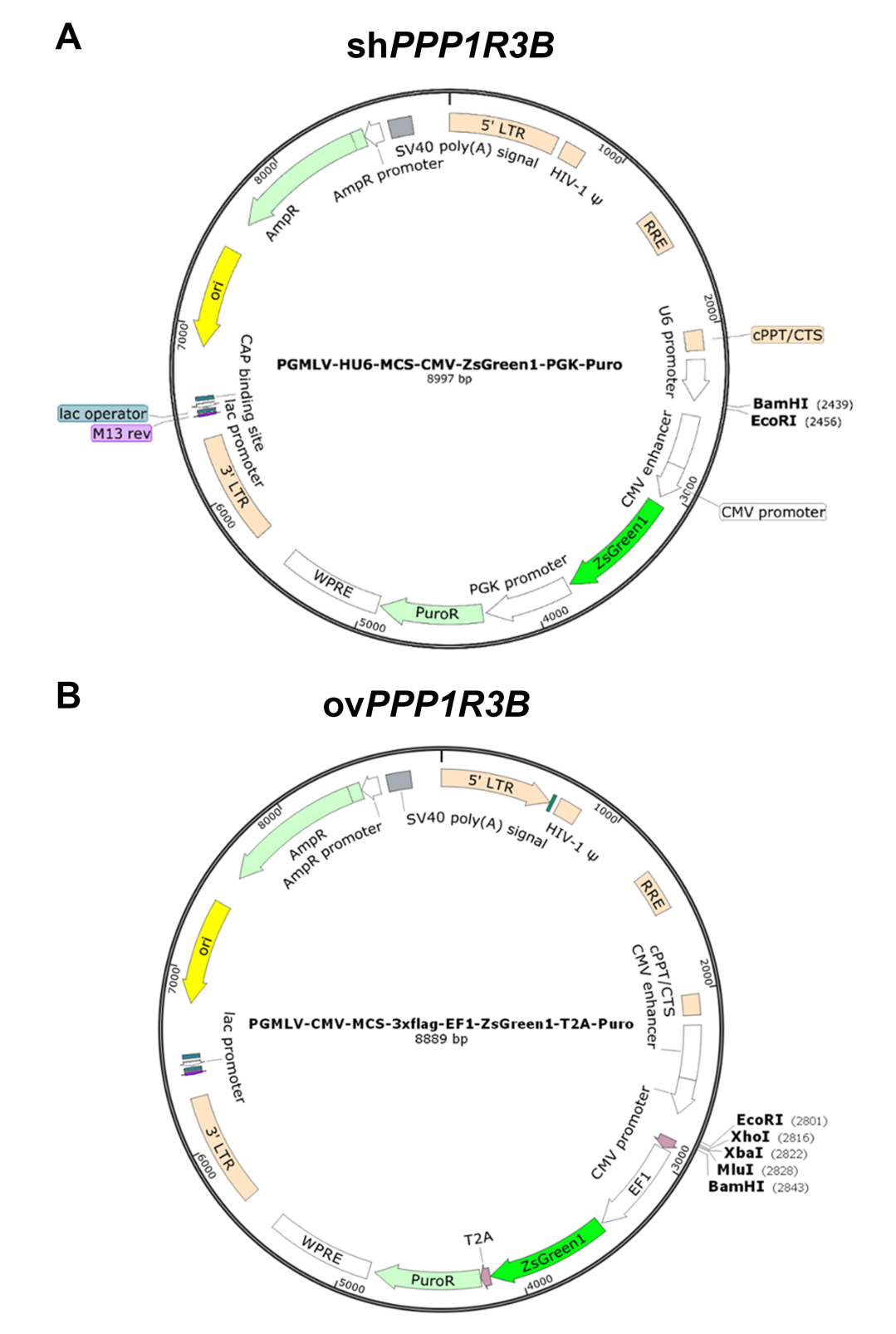


**Supplementary Fig. 5.** Schematic diagrams of viral vectors **(A)** overexpressing and **(B)** knocking down *PPP1R3B*.

**
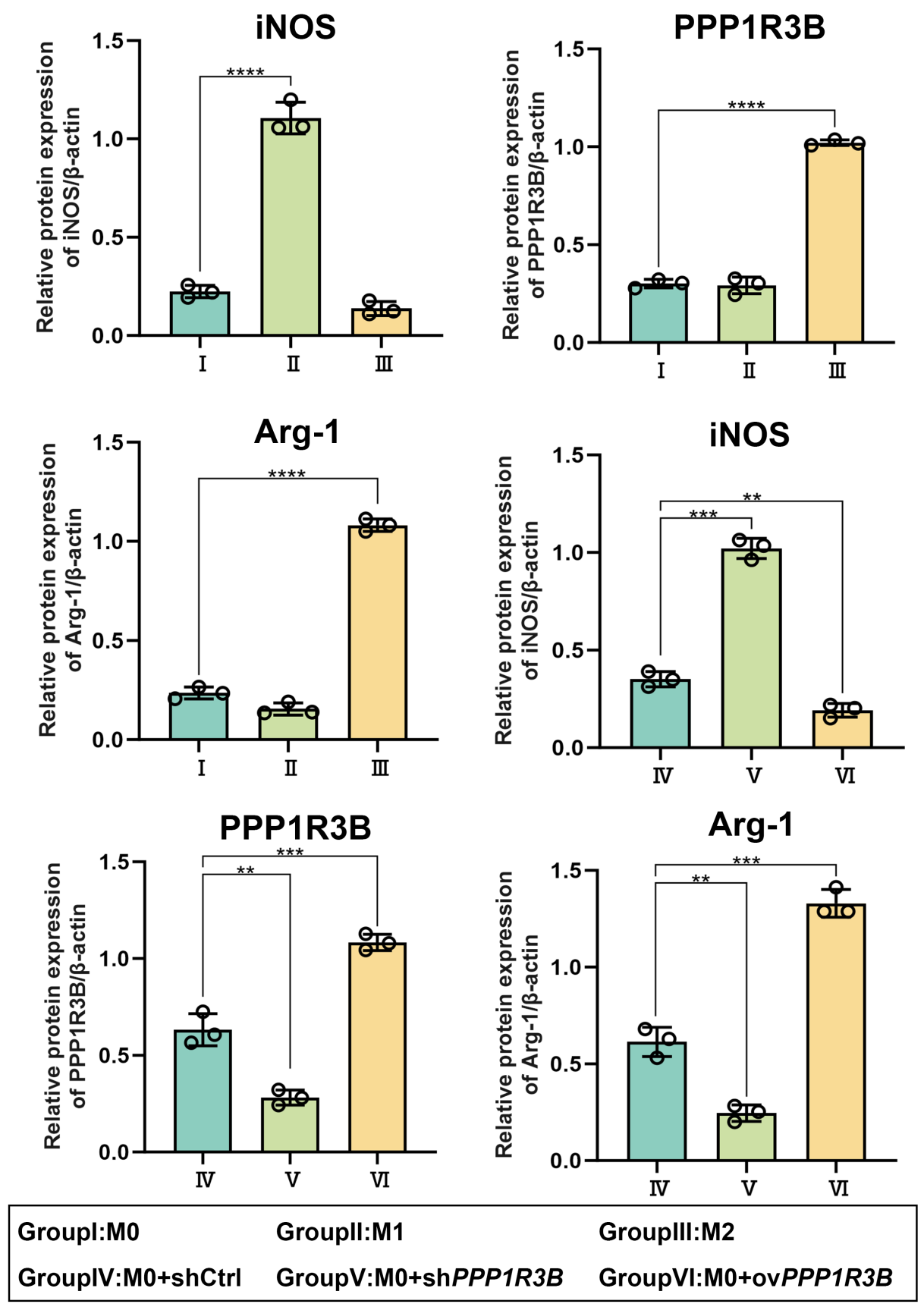
**

**Supplementary Fig. 6.** Statistical histogram of (Fig. 2A).The data are presented as the mean ± SEM across three biologically independent samples. Significance: **p* < 0.05; ***p* < 0.01; ****p* < 0.001; *****p* < 0.0001.


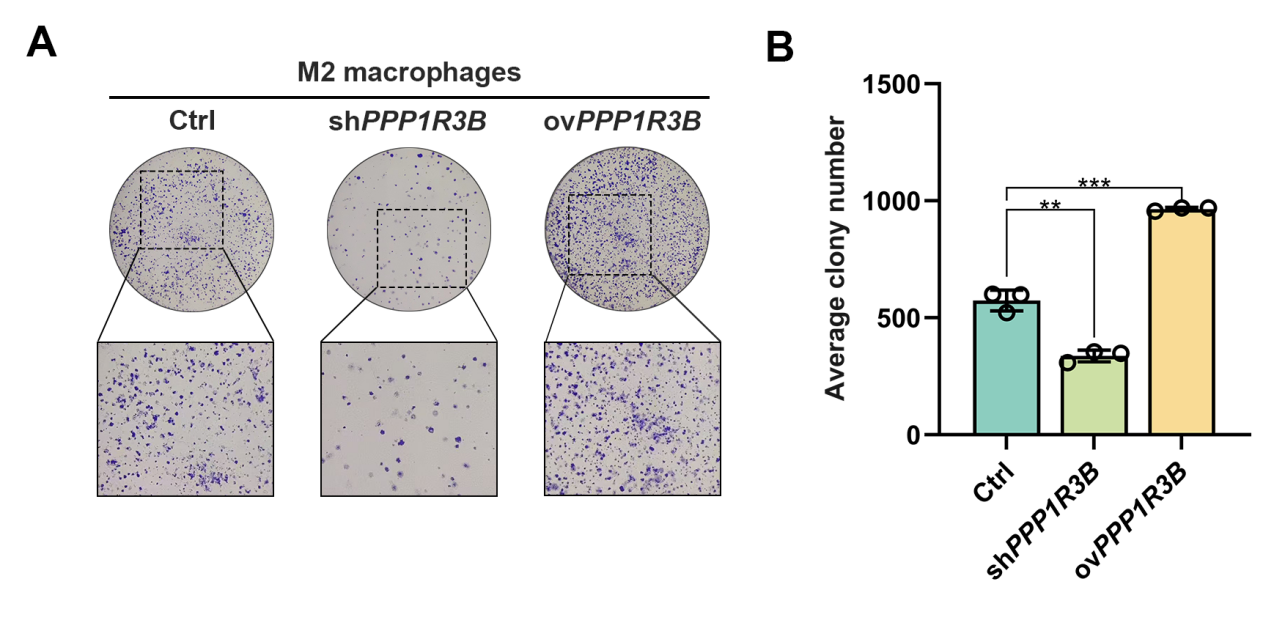


**Supplementary Fig. 7. (A)** Representative images of colony formation showing *PPP1R3B* regulation of M2 MΦs proliferation. **(B)** Statistical histogram of (A). The data are presented as the mean ± SEM across three biologically independent samples. Significance: **p* < 0.05; ***p* < 0.01; ****p* < 0.001; *****p*< 0.0001.


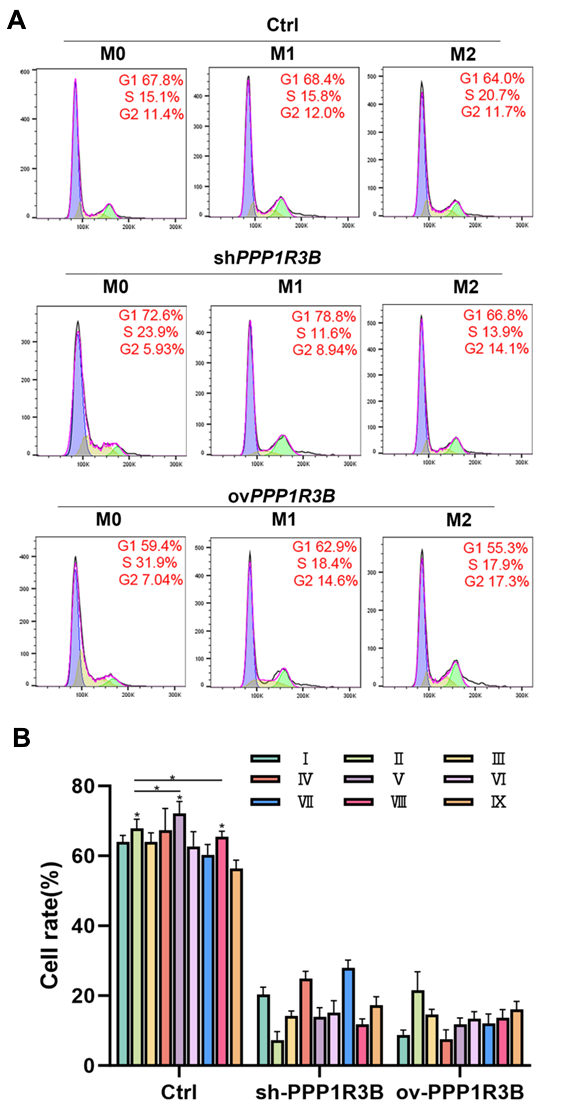
**Supplementary Fig. 8.** **(A)** Representative flow cytometric cell cycle images of MΦ regulated by *PPP1R3B*. **(B)** Statistical histogram of (A). The data are presented as the mean ± SEM across three biologically independent samples. Significance: **p* < 0.05; ***p* < 0.01; ****p* < 0.001; *****p*< 0.0001.


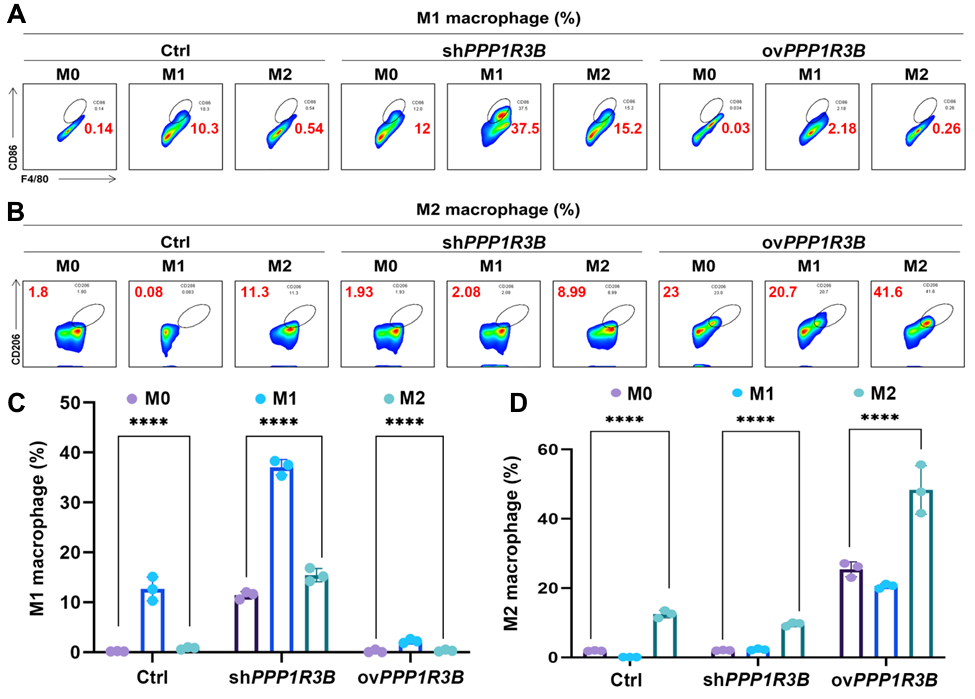


**Supplementary Fig. 9.** Flow cytometry identified **(A)** M1 and **(B)** M2 MΦs, marked by adhesion G protein-coupled receptor E1 (ADGRE1/F4/80^+^) with CD86^+^ for M1 and CD206^+^ for M2, and a quantitative assessment of the **(C)** M1 and **(D)** M2 population after various treatments. The data are presented as the mean ± SEM across three biologically independent samples. Significance: **p* < 0.05; ***p* < 0.01; ****p* < 0.001; *****p* < 0.0001.


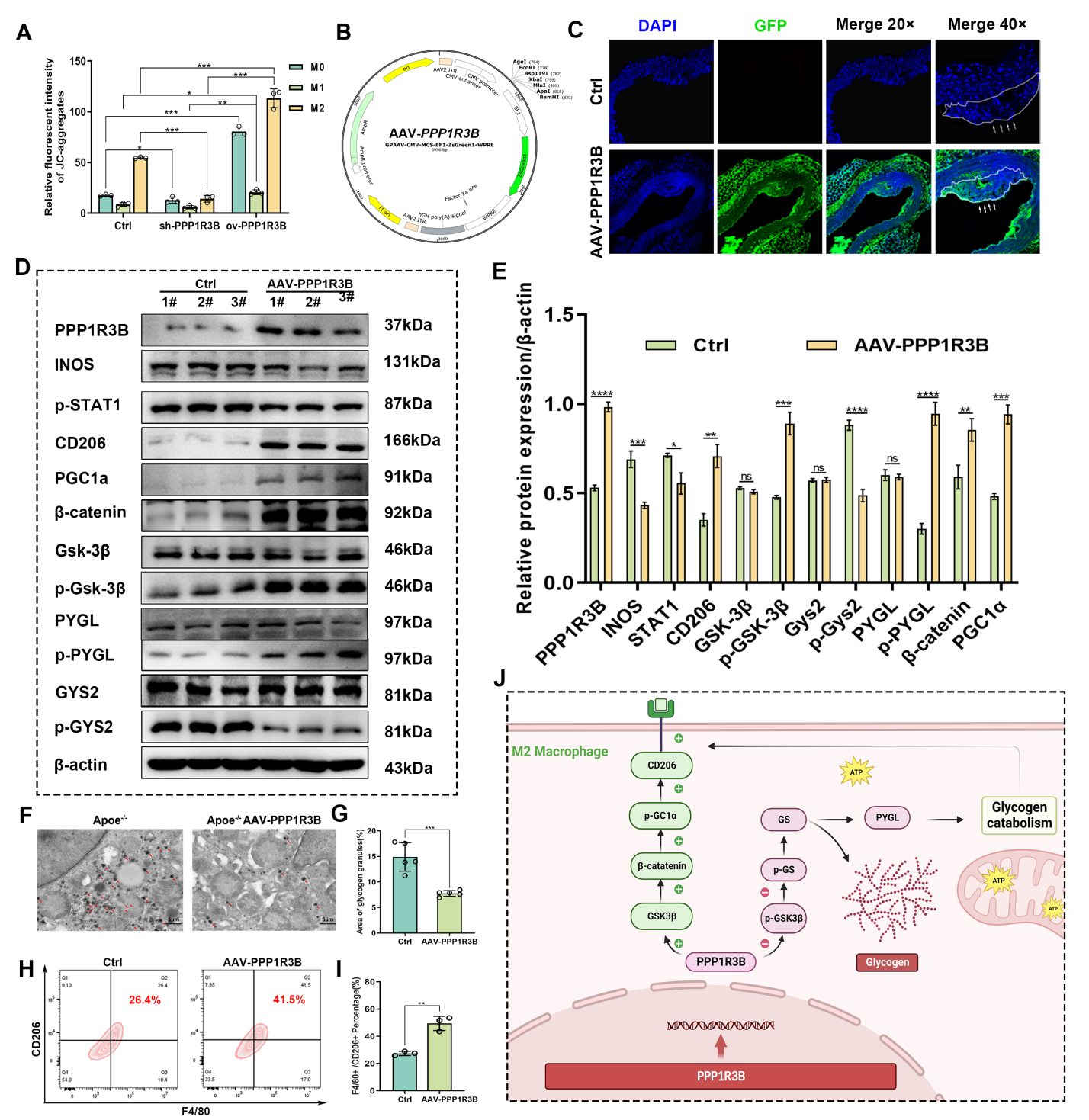


**Supplementary Fig. 10**. **(A)** Statistical histogram of Figure 3C. **(B)** Schematic diagram of the AAV-*PPP1R3B* vector. **(C)** Immunofluorescence staining illustrates the transfection efficiency of AAV-*PPP1R3B* in aortic plaques in *Apoe*^−/−^ mice. **(D)** Western blot of aortic plaque suspensions from *Apoe*^−/−^ mice treated with the control and AAV-*PPP1R3B* vectors. **(E)** Statistical histogram of (D). **(F)** Representative TEM images of glycogen granules in the aortic cross-sections of *Apoe*^−/−^ mice from the control and AAV-*PPP1R3B* groups. **(G)** Statistical histogram of (F). **(H)** Flow cytometry of aortic plaque suspensions from *Apoe*^−/−^ mice treated with control or AAV-*PPP1R3B* vectors. **(I)** Statistical histogram of (H). **(J)** Schematic diagram of *PPP1R3B* counteracting AS by promoting M2 MΦ polarization and glycogen metabolism reprogramming. The data are presented as the mean ± SEM across three biologically independent samples. Significance: **p* < 0.05; ***p* < 0.01; ****p* < 0.001; *****p*< 0.0001.


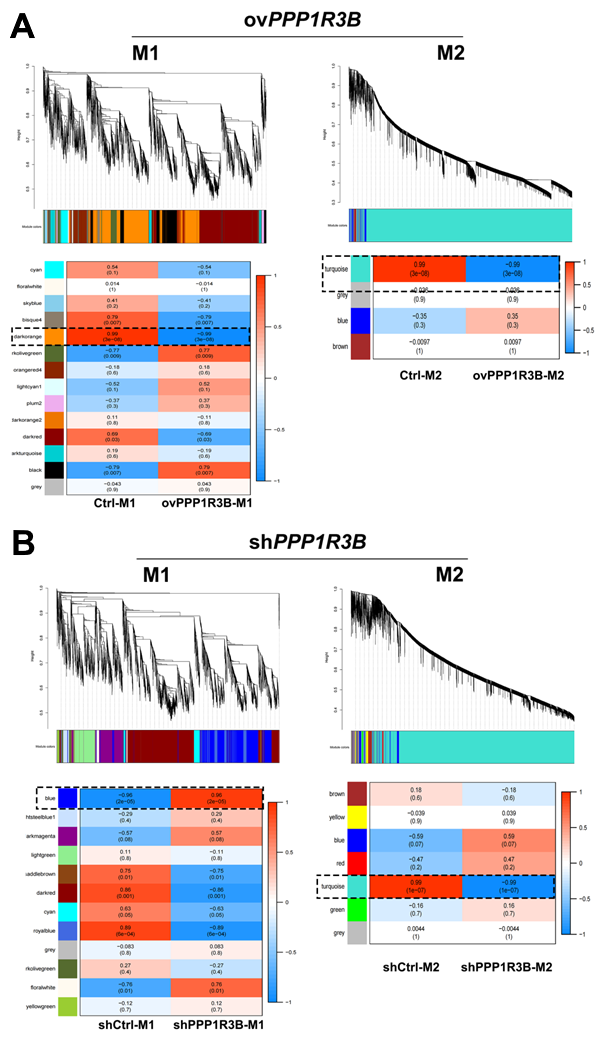


**Supplementary Fig. 11**. WGCNA of RNA-seq data for M1/M2 MΦs treated with **(A)** ov*PPP1R3B* and **(B)** sh*PPP1R3B*.


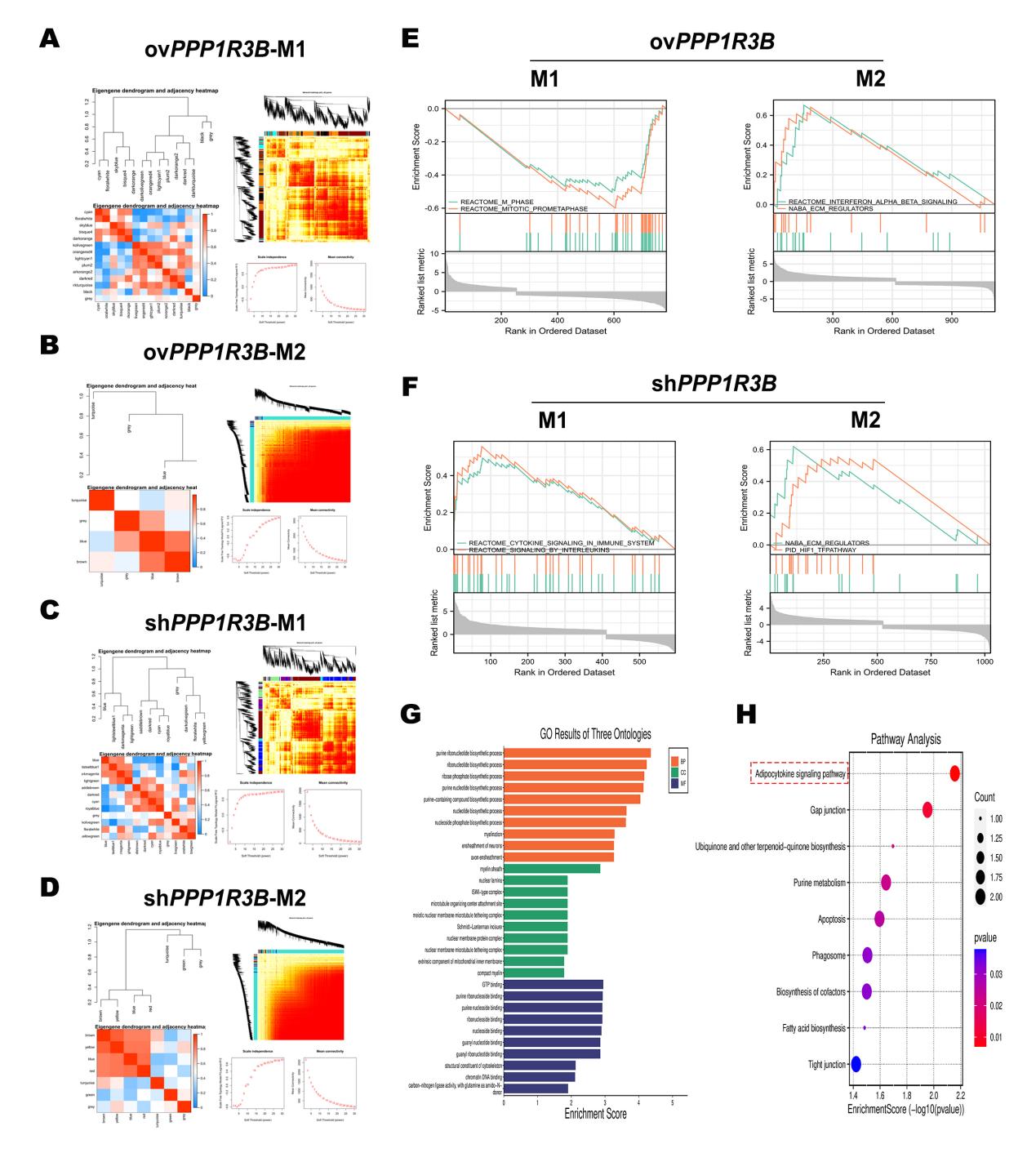


**Supplementary Fig. 12**. WGCNA of **(A)** ov*PPP1R3B*-M1, **(B)** ov*PPP1R3B*-M2, **(C)** sh*PPP1R3B*-M1, and **(D)** sh*PPP1R3B*-M2. GSEA of **(E)** ov*PPP1R3B* and **(F)** sh*PPP1R3B* in M1 and M2 MΦs. **(G)** Histogram and **(H)** bubble chart of GO enrichment of MΦ immune metabolism-related DEGs regulated by *PPP1R3B.*


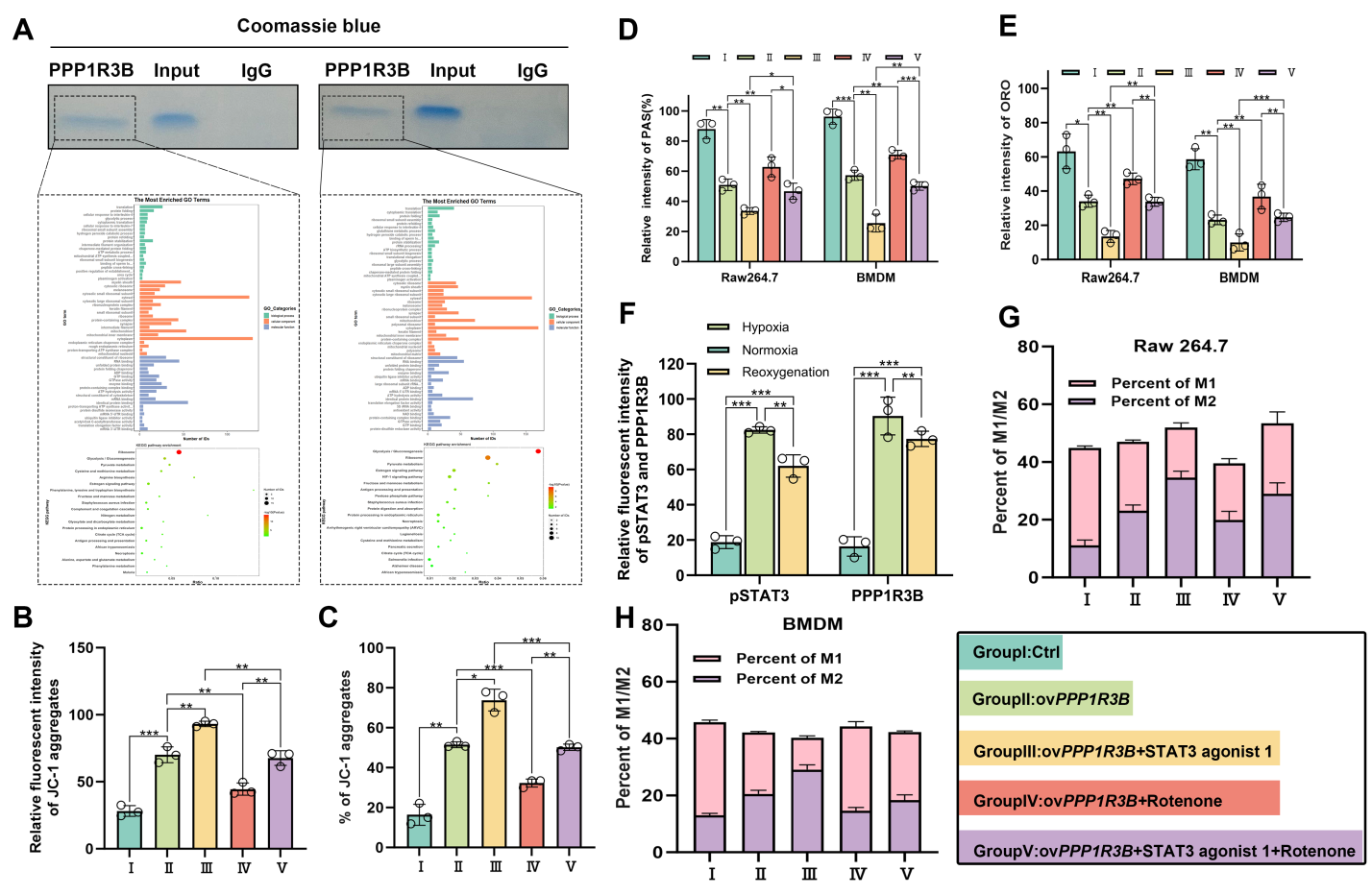


**Supplementary Fig. 13**. **(A)** IP-MS analysis of proteins interacting with *PPP1R3B*. **(B)** Statistical histogram of JC-1 staining in Figure 5E. **(C)** Statistical histogram of JC-1 flow cytometry in Figure 5F. **(D, E)** Quantitative statistical histogram of ORO staining and PAS staining in Figure 5G. **(F)** Quantitative statistical histogram representation of pSTAT3 and PPP1R3B expression under various conditions in immunofluorescence assays in Figure 5H. Quantitative statistical histogram representation of flow cytometry analysis in **(G)** RAW264.7 MΦs and **(H)** BMDMs. The data are represented as the mean ± SEM across three biologically independent samples. Significance: **p* < 0.05; ***p* < 0.01; ****p* < 0.001; *****p* < 0.0001.


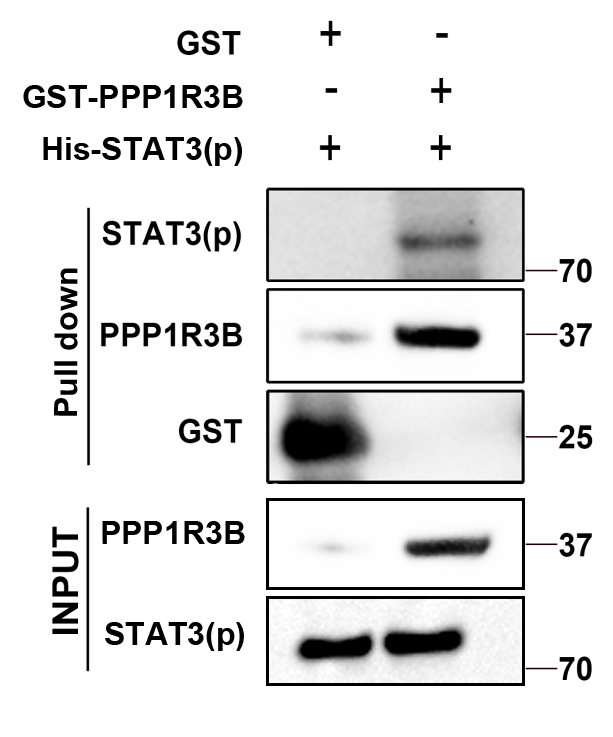


**Supplementary Fig. 14.** GST pull-down assay demonstrating the interaction between *PPP1R3B* and phosphorylated STAT3 (p-STAT3). Western blot analysis of pull-down fractions revealed specific binding of p-STAT3 to GST-*PPP1R3B*. Inputs confirm the presence of *PPP1R3B* and p-STAT3 used in the assay.


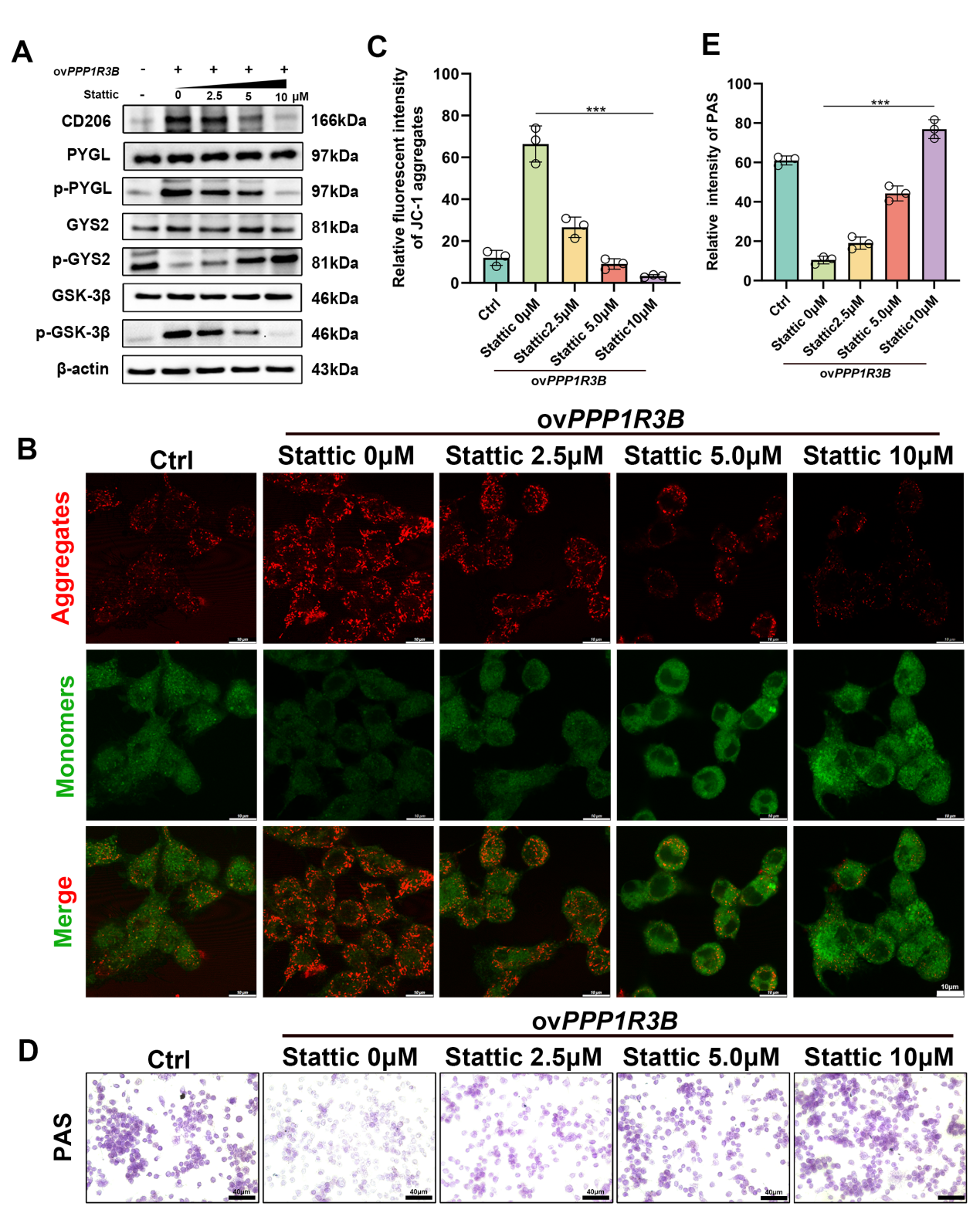


**Supplementary Fig. 15**. **(A)** Western blot analysis of glycogen metabolism and macrophage phenotype following STAT3 inhibition with a dose gradient of Stattic (Calbiochem, San Diego, CA, USA). **(B)** Different concentrations of STAT3 inhibitor (Stattic) groups have representative JC-1 fluorescence staining images. **(C)** Quantitative statistical histogram of (B). (D) Representative PAS staining images in the different concentrations of STAT3 inhibitor (Stattic) groups. **(E)** Quantitative statistical histogram of (D). The data are presented as the mean ± SEM across three biologically independent samples. Significance: **p* < 0.05; ***p* < 0.01; ****p* < 0.001; *****p* < 0.0001.


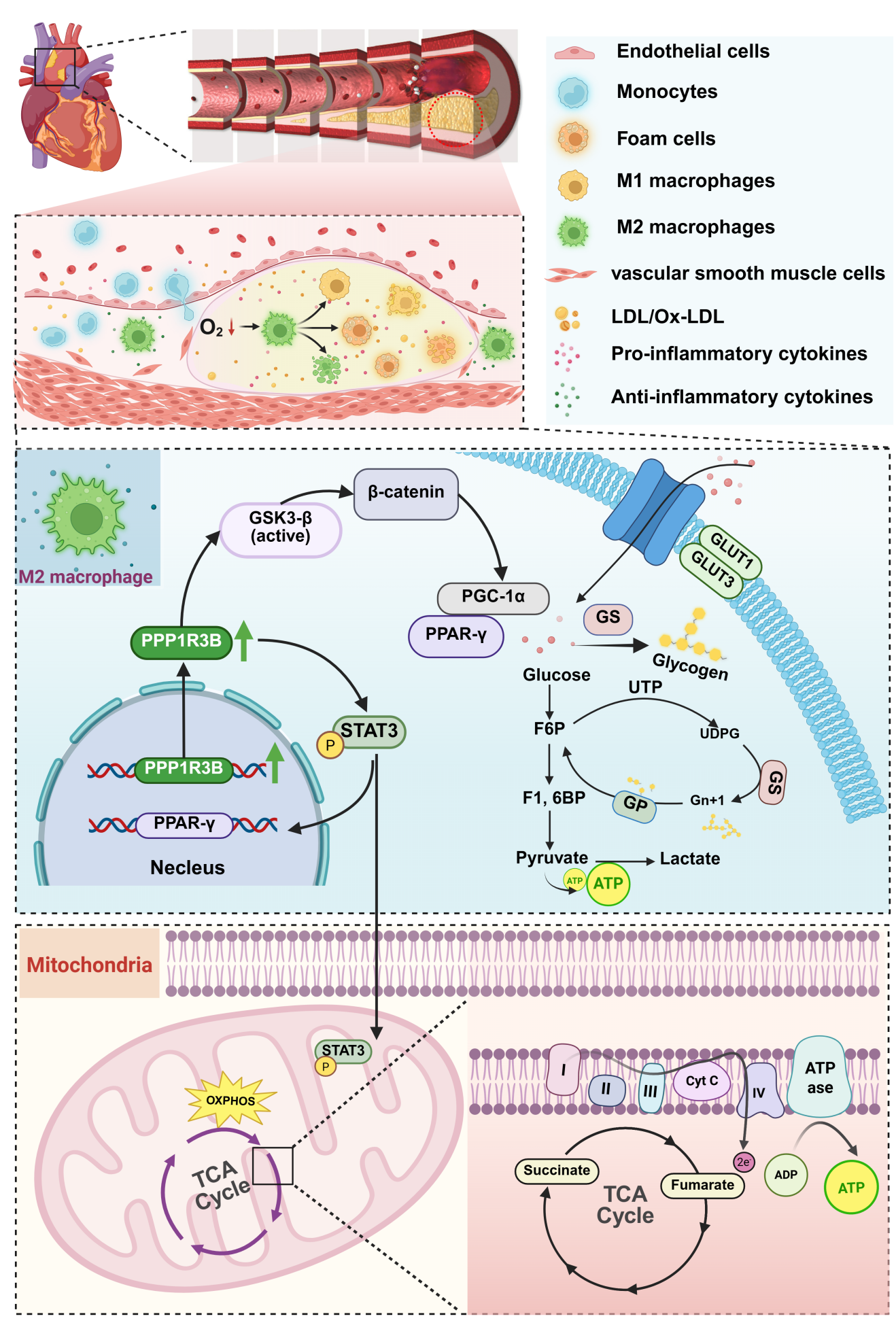


**Supplementary Fig. 16**. The mechanism of *PPP1R3B* binding and activating to phosphorylate STAT3. *PPP1R3B*-activated p-STAT3 promotes the expression of *MT-CO1* and *MT-CO3*, pivotal components of mitochondrial energy metabolism, thereby enhancing the degradation and energy supply derived from glycogen and lipids within mitochondria.


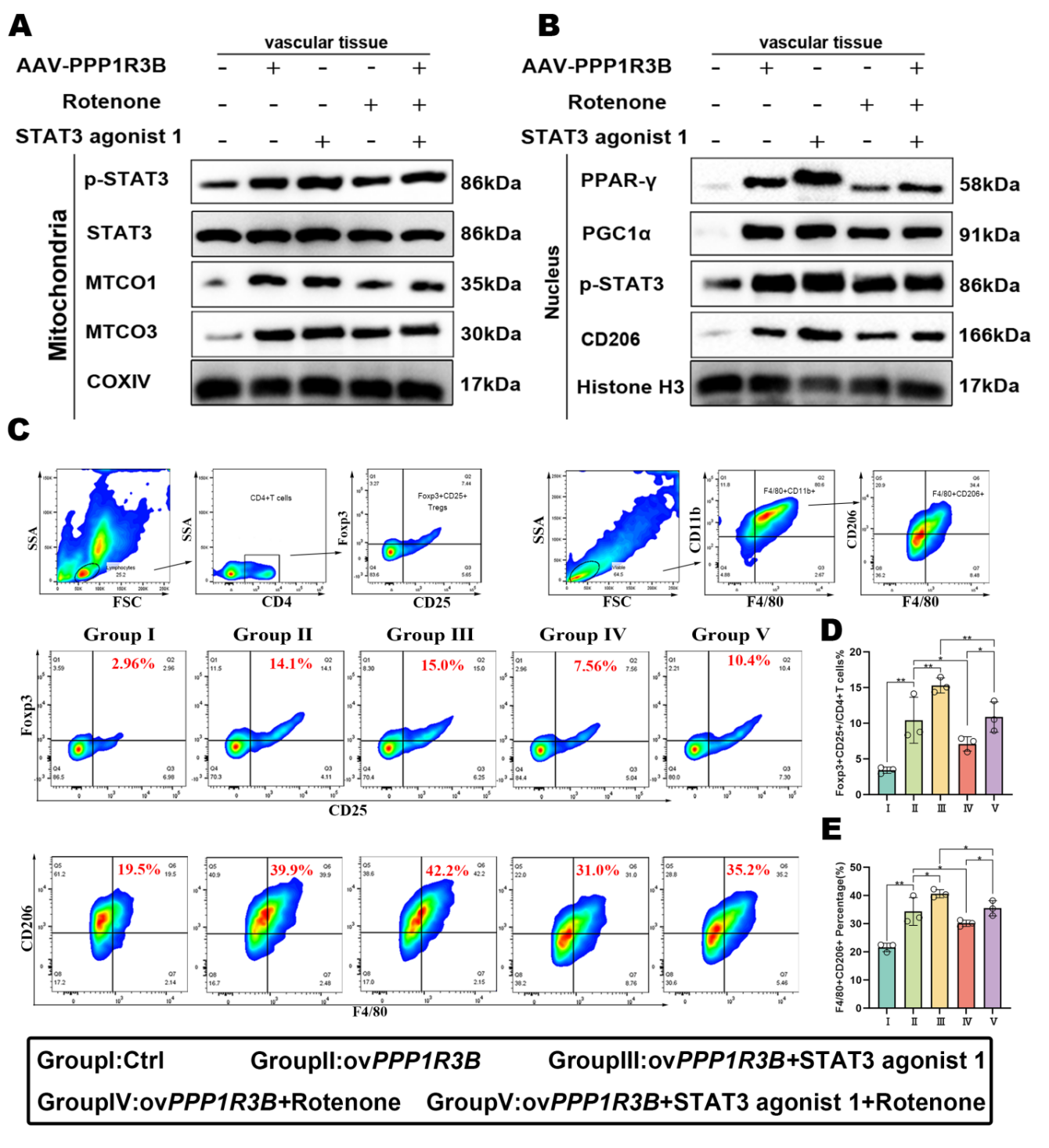


**Supplementary Fig. 17**. Western blots of **(A)** mitochondrial and **(B)** nuclear proteins from *Apoe*^−/−^ mice in each treatment group. **(C)** Flow cytometry analysis of Tregs (FOXP3^+^ and CD25^+^) and M2 (F4/80^+^ and CD206^+^) from *Apoe*^−/−^ mice in each treatment group. Quantitative statistical histograms of **(D)** Tregs and **(E)** M2 MΦs from (C). The data are presented as the mean ± SEM across three biologically independent samples. Significance: **p* < 0.05; ***p* < 0.01; ****p* < 0.001; *****p* < 0.0001. Treatment groups: I, control; II, AAV-*PPP1R3B*; III, AAV-*PPP1R3B*+STAT3 agonist 1; IV, AAV-*PPP1R3B*+rotenone; V, AAV-*PPP1R3B*+STAT3 agonist 1+rotenone.
